# Despite misinformation, low trust, and conflict in Somalia, high demand for vaccines and a negative endorsement effect of non-state authorities

**DOI:** 10.1038/s41598-023-48389-7

**Published:** 2023-12-07

**Authors:** Laurits F. Aarslew, Nicholas Haas, Prabin B. Khadka

**Affiliations:** 1 Department of Political Science, Aarhus University, Aarhus, Denmark; 2 Department of Government, University of Essex, Colchester, UK

**Keywords:** Human behaviour, Psychology

## Abstract

Expanding vaccination coverage in conflict regions is crucial to the prevention of further mutations and outbreaks of COVID-19, as well as of future pandemics, worldwide. However, scholars’ suppositions that low levels of trust and control over the flow of misinformation in these regions may necessitate tailored solutions—in particular, that health advice come from trusted non-state authorities—remain largely untested. To better understand the levels and drivers of COVID-19 vaccine receptivity in conflict regions, we partnered with the United Nations and the Somali Ministry of Justice to field a three-wave panel survey ($N_1$ = 1672; $N_2$ = 880; $N_3$ = 908) and vaccine endorsement experiment in South Central Somalia. We observe high overall demand for vaccines (between 90 and 94%), particularly among those who have experienced violence and illness, who perceive high economic disruption due to the pandemic, and who report more favorable views of and exposure to the West and Western-affiliated organizations. The high overall demand is particularly striking given respondents’ low interpersonal trust and considerable exposure to vaccine disinformation. Contrary to scholars’ and policymakers’ expectations, we find that whereas endorsements from government health authorities increase vaccine receptivity, endorsements from non-state customary authorities and extremist militant group Al-Shabaab decrease support for vaccination (− 8 and − 16% points, respectively). Our findings caution against presupposing a need for different approaches to increasing vaccination coverage in conflict regions, and suggest that at least in the short-term, constraints on vaccine supply and access are more likely to bind than those on demand.

## Introduction

What are the levels and drivers of COVID-19 vaccine hesitancy and acceptance in conflict regions? As the supply of vaccines expands, understanding vaccine demand and its determinants around the world will become critical to efforts both to close the vaccine equity gap and to prevent the emergence of new disease variants^1^. However, many of the countries with the lowest vaccination rates are also the ones about which we possess the least evidence, often because violence impedes the collection of high-quality data^2^. Research expanding our knowledge of vaccine hesitancy and acceptance in conflict-ridden regions has thus been highlighted as a priority, not only to inform responses to the current global pandemic of COVID-19, but also to future pandemics and disease eradication efforts^3, 4^.

Deriving expectations about vaccine demand and its drivers in conflict regions is not a straightforward task. On the one hand, studies finding comparatively high vaccine demand in low-income countries suggest that acceptance should be high in conflict-ridden countries, which typically rank among the world’s most impoverished^5–7^. On the other hand, political instability likely puts conflict regions at a disadvantage through two factors that have proven critical to informing COVID-19 outcomes in much of the world: interpersonal and government trust and the prevalence of misinformation^3, 8–11^. Drawing on these latter two deficits, many scholars and policymakers have accordingly anticipated both low overall vaccine demand and a need for tailored solutions in such regions^3, 12–15^. One particularly popular proposed solution is the greater involvement of non-state and customary authorities as vaccine and public health advocates^3, 12^. For instance, scholars in a recent article write, “Partnering in advance with local cultural, ethnic, or faith-based institutions and leaders in divided communities can help identify trusted messengers and improve communication and response when the next health crisis arises”^3^. These competing expectations and associated approaches call for further exploration of vaccine receptivity in conflict regions.

In this paper, we aim to increase knowledge about vaccine demand and its drivers in South Central Somalia, a region that ranks among both the most conflict-prone and least vaccinated in the world^4, 16, 17^. Violence has long hampered data collection in Somalia, forcing researchers hoping to study health outcomes in the country to rely on “fragmentary survey data” that often only provides proxies for their central measures of interest^2^. To overcome these historic barriers, we partnered with the United Nations Development Programme Rule of Law Somalia Office (UNDP RoL Somalia) and with the Somali Ministry of Justice. We collected three rounds of panel survey data between January 2021 and April 2022 from across seven cities and with between 880 and 1672 respondents in each wave. We collected data in three waves by inviting a randomly chosen subset of respondents to our first survey to participate in our second and third surveys. Our panel design allows us to evaluate whether and how views on vaccines changed over time; importantly, the surveys cover periods when vaccines were not yet available and vaccination was merely hypothetical, as well as when they became more readily accessible and the choice was more concrete.

In addition to better understanding vaccine hesitancy and its correlates in the Somali context, we also sought to evaluate different strategies to increase vaccine demand. To this end, we embedded an endorsement experiment into our surveys to probe if explicit support for vaccination efforts from different salient political actors affected individuals’ own support for vaccination campaigns. Specifically, we asked respondents whether they agreed with a statement—that everyone in Somalia should be vaccinated against COVID-19—which, depending on a respondent’s randomly assigned treatment, had been endorsed either by no one (baseline condition) or by a salient political actor: either the domestic government, international organizations, extremist militant group Al-Shabaab, or customary authorities (clan and religious elders). We elicit support specifically for a “COVID-19” vaccination campaign, as this was our primary outcome of interest and we were confident that respondents were aware of the pandemic and associated vaccine. Consistently, a World Bank telephone survey carried out in Somalia to measure the socio-economic impact of COVID-19 shows that 99.23% (*N* = 2,744) of respondents replied “Yes” when asked, “Have you heard about the COVID-19 or the pandemic or epidemic associated with the coronavirus?”^18^. We use results from our endorsement experiment to investigate whether appeals from certain political actors might be more or less effective at increasing vaccine demand. Critically, the experiment allows us to evaluate whether, as has been posited by many policymakers and scholars, religious, ethnic, and non-state armed militant leaders should be viewed as more persuasive vaccine messengers than the state in conflict and low-trust regions^3, 12–15^. Notably, while most policymakers and scholars have focused on religious, ethnic, and cultural authorities as possible vaccine messengers, some have explicitly called for partnerships with militant groups. For instance, a legal and policy advisor at a large nongovernmental organization was recently quoted as saying that in conflict regions, “in order to solve COVID-19-related problems, humanitarian actors should engage with the authorities of these armed nonstate actors on the distribution of the vaccine”^12^.

## Results

**Figure 1:**
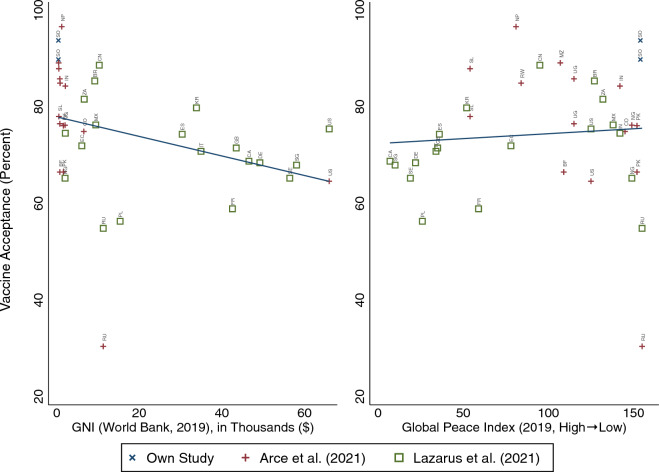
Vaccine acceptance in comparative perspective. This figure displays vaccine acceptance across countries; we include our own estimates for Somalia across two waves, as well as estimates reported in two additional studies^7, 22^. We explore relationships between vaccine acceptance and pre-pandemic gross national income (left panel)^23^ and peace (right panel)^24^. Our vaccine acceptance data was collected in January–February 2021 (wave 1) and August 2021 (wave 2), as compared with June 2020–January 2021^7^ and June 2020^22^. A full list of all countries, their estimated vaccine acceptance by study, and their 2019 GNI and GPI, can be found in SI Table A1. Regression output and results using alternative measures to GPI for exposure to conflict can be found in SI Sect. B.

**Figure 2:**
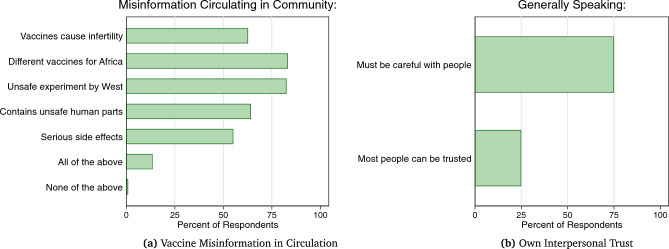
Misinformation and trust in Somalia. Left panel displays the percentage of survey 2 respondents reporting that they had (**a**) heard each listed piece of vaccine misinformation in circulation in the community and (**b**) which they thought had made at least some people hesitant to take vaccines in their community. Right panel displays the percentage of the same respondents stating that, generally speaking, they believed either that one “must be very careful in dealing with people” or that “most people can be trusted”.

**Figure 3:**
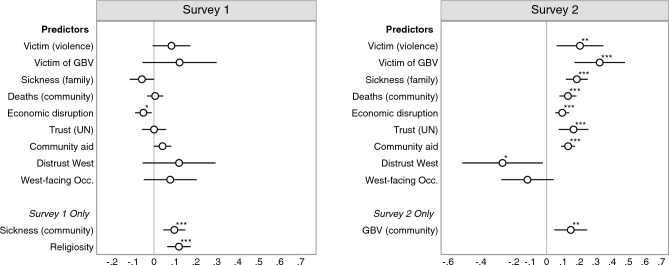
Covid-19 vaccine receptivity predictors. Estimates based on unstandardized OLS regressions with city fixed effects, controlling for treatment assignment. Whiskers are 95% confidence intervals based on robust standard errors clustered on communities. *$p<0.05$, **$p<0.01$, ***$p<0.001$ (two-sided tests).

**Figure 4:**
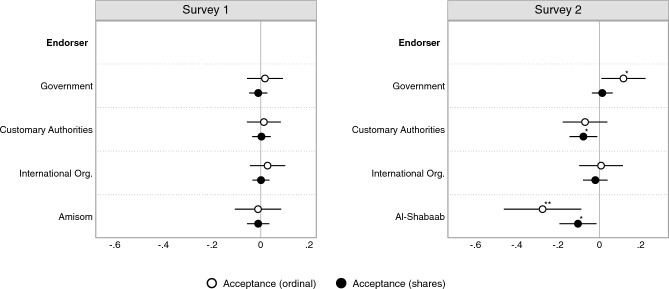
Endorsement experiment. This figure displays the difference in vaccine acceptance between the baseline group and the endorsement groups, estimated for both the ordinal acceptance scale (white dots) and acceptance shares (black dots). Dots are point estimates and bars are 95% confidence intervals. Estimates are based on unstandardized OLS regressions with city fixed effects and robust standard errors clustered on communities. *$p<0.05$, **$p<0.01$, ***$p<0.001$ (two-sided tests).

**Figure 5:**
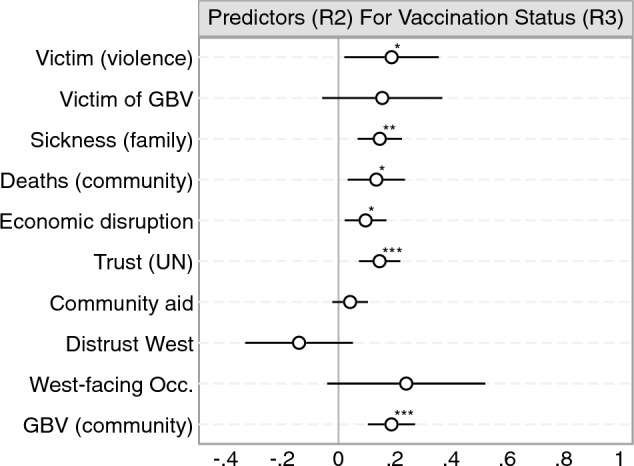
Covid-19 vaccine take-up predictors. Estimates based on unstandardized OLS regressions with city fixed effects. Whiskers are 95% confidence intervals based on robust standard errors clustered on communities. *$p<0.05$, **$p<0.01$, ***$p<0.001$ (two-sided tests).

We begin by comparing levels of vaccine hesitancy in Somalia to levels in other regions. Next, we investigate predictors of vaccine hesitancy and present results from our endorsement experiment to evaluate which endorsers, if any, are able to increase demand for vaccines—and if so, among whom. Lastly, we examine the correlates of vaccine take-up. Consistent with our pre-analysis plan, we present descriptive statistics as well as results from OLS regressions including city fixed effects and clustering standard errors at the community level. We discuss data collection and estimation strategy at greater length in our “Methods” section, and we present additional analyses and robustness tests in our Supplementary Information SI section. In the interest of simplicity, we present our main findings in Figs. 1, 2, 3, 4 and 5. All accompanying regression tables can be found in the supplementary material (SI Sect. D).

### Characteristics of the sample and the experiment

Our study relies on a three-wave panel survey, with an embedded endorsement experiment, conducted in Somalia between January 30, 2021, and April 2, 2022. The data was collected in collaboration with the United Nations Development Programme Rule of Law Somalia Office (UNDP RoL Somalia). We collected our data in three waves by inviting a randomly chosen subset of respondents from our first survey to participate in the second and third survey waves (the “Methods” section describes our data collection procedure in detail).

To examine the effects of COVID-19 vaccine endorsements, we randomly assigned participants to one of five experimental conditions (four treatment groups and a baseline group). In the first and second surveys, participants were all asked about their support for the statement that “everybody should get vaccinated.” In the four treatment conditions, the statement was endorsed by a specific source (Survey 1: the government, customary authorities, international organizations, or the African Union Mission to Somalia [AMISOM]; Survey 2: the government, customary authorities, international organizations, or Al-Shabaab). The baseline group did not receive any endorsement and was only asked its agreement with the statement. The exact question wording in survey 2 was as follows (only the text in italics varied between experimental conditions):

“Now that the COVID-19 vaccine is available in your community, ([*SOURCE*] *believes that*) everyone should be vaccinated. Do you agree with this statement that everyone should be vaccinated?”

Participants marked their responses on a scale from 1 (strongly disagree) to 4 (strongly agree) (Survey 1: M = 2.95, SD = 0.58; Survey 2: M = 3.08, SD = 0.66). In the third survey (without an embedded experiment), participants were asked “did you take the COVID-19 vaccine?” Participants could mark one of four options (“no”, “yes, but only one dose”, “yes - two doses”, “yes - three doses (two + booster dose)”, or a don’t know option). Of the 873 respondents to provide an answer in the third survey, 49.9% (n = 436) were not vaccinated, 36.8% (n = 322) were vaccinated with a single dose, 11.1% (n = 97) were vaccinated with two doses, and 1.8% (n = 16) were vaccinated with two doses and a booster dose (D/K = 0.23%). As of October 2022, an estimated 30% of the population in Somalia was said to be fully vaccinated against COVID-19^19^. The “Methods” section presents additional information on all relevant variables in our analysis; SI Sect. A contains links to our survey questionnaires.

### Somalia in comparative perspective

Does vaccine hesitancy differ in Somalia as compared with other regions? Based on existing scholarship, we expect vaccine acceptance rates to be comparatively higher in low-income countries^6, 7^. Possible proposed reasons for this relationship are many and include greater economic disruption due to COVID-19 in poorer countries and accordingly a stronger desire to return to normalcy, greater pre-COVID-19 acceptance of childhood vaccination in these regions, and differences in information provision across regions^7, 20, 21^. Given that more conflict-prone countries tend to be poorer, we thus might anticipate that countries experiencing more conflict will be more receptive to vaccines^5^. However, there are also reasons to believe that more conflict-ridden countries will be more vaccine-hesitant. Notably, such regions are often characterized by low trust and a high degree of misinformation^9, 12–15^ (see also Fig. 2). Additionally, individuals may fear retaliation from militant groups, and one might surmise that the perceived risk of COVID-19 relative to other daily risks is lesser than in more peaceful contexts^9^.

Figure 1 compares vaccine hesitancy across two waves of our survey in Somalia to hesitancy as reported in two global studies^7, 22^. We distinguish between studies and display country-level correlations between vaccine acceptance and pre-pandemic (2019) gross national income^23^ (left panel) and ranking in a global peace index^24^ (right panel). The findings are similar when using non-ranked alternative measures for exposure to conflict (see SI Sect. B). A few observations are worth note. First, we observe that vaccine receptivity in Somalia is very high in absolute terms (90% in wave 1 and 94% in wave 2, see Appendix Table A1). Reassuringly, we observe a similarly high level of vaccine receptivity in the aforementioned World Bank telephone survey measuring the socio-economic impact of COVID-19 in Somalia: they observe a reported acceptance rate of approximately 90% (93% when limited to the same seven urban areas as in our study)^18^ (see SI Sect. K.3). Second, we see that despite these high absolute numbers, vaccine acceptance in Somalia is nevertheless at a similar level as in a number of other countries: notably, Nepal (97%), Mozambique (89%), China (89%), Sierra Leone (88%), and Uganda (86%). Third, as expected, we observe a negative, albeit weak, correlation between country income and vaccine acceptance, but no evidence of a correlation between peace and acceptance. The country income correlation is not robust to the inclusion of country and study fixed effects (see SI Table A2). Fourth, we find suggestive evidence that vaccine acceptance increased from wave 1 to wave 2 of our survey, which would be consistent with over-time findings from a number of other regions around the world^20^. A two-sample test of proportions, however, indicates that acceptance across the two waves is not statistically distinguishable from zero ($p=0.06$ using a one-tailed test, and $p=0.14$ using a two-tailed test).

As noted earlier, scholars have posited that conflict regions featuring low trust and a high prevalence of vaccine misinformation should exhibit lower overall demand for vaccines. It is thus worth noting that both of these characteristics are present in our sample of respondents in South Central Somalia, as can be seen in Fig. 2. It therefore appears that high vaccine demand can exist, at least in the Somali context, alongside low trust and high exposure to misinformation.

### Correlates of vaccine acceptance

Next, we analyze a series of predictors for vaccine acceptance using the first and second surveys. Figure 3 displays correlations between a range of individual and community-level covariates and vaccine acceptance (using the four-point ordinal scale). These are estimated using OLS regression functions, controlling for endorsement treatment assignment and including city fixed effects, with robust standard errors clustered at the community level. In the supplementary material, we show that the findings in Fig. 3 are robust to adjusting for additional covariates and restricting the sample to the same set of respondents across the two survey rounds (SI Sects. E and F).

Figure 3 shows that a series of covariates strongly predict vaccine acceptance but only in the second survey. We speculate that the unavailability of COVID-19 vaccines at the time of fielding survey 1 may explain the comparative lack of detectable differences at that stage. As COVID-19 vaccines became available around the time of the second survey’s fielding, we conjecture that participants provided more careful and informed answers to questions about vaccine acceptance at that point. Indeed, studies have documented within-respondent changes in vaccine receptivity and its predictors over time^25^, and scholars have similarly speculated that for surveys conducted when “no COVID-19 vaccine was publicly available...stated acceptance is hypothetical and may change with provision of more information about current vaccines”^26^. For survey 2, Fig. 3 shows that victims of overall violence and gender-based violence display greater vaccine acceptance ($\hat{\beta } = 0.20$; $p =$ 0.006; Cohen’s D = 0.39; 95% CI [0.06, 0.35] and $\hat{\beta }$ = 0.32; $p<$ 0.001; Cohen’s D = 0.63; 95% CI [0.17, 0.48], respectively). We use the standard deviation of the vaccine acceptance measure in the baseline group to calculate Cohen’s D. All three measures of COVID-19 impact (family sickness, COVID-19 related deaths in the community, and economic disruption caused by COVID-19) are positively associated with vaccine acceptance ($\hat{\beta }$ = 0.18; Cohen’s D = 0.36; 95% CI [0.11, 0.25]; $\hat{\beta }$ = 0.13; Cohen’s D = 0.25; 95% CI [0.07, 0.18]; and $\hat{\beta }$ = 0.09; Cohen’s D = 0.25; 95% CI [0.05, 0.14], respectively). All three coefficients are significant at the 0.1% level ($p<$ 0.001). Respondents who report having greater trust in the UN and living in a community that has received more community aid are also more likely to agree that everybody should get vaccinated. Respondents who express distrust of the West also display greater vaccine hesitancy ($\hat{\beta } = -0.27$; $p = 0.032$; Cohen’s D = 0.25; 95% CI [$-0.52, -0.02$]); however, participants who work in West-facing occupations (government jobs or NGOs/INGOs) do not express greater vaccine acceptance.

### Endorsement experiment

Next, we turn our attention to examining the impact of COVID-19 vaccination endorsements on vaccine receptivity in the between-subject experiment described above. Figure 4 displays the estimated difference in means from unstandardized OLS regressions with city fixed effects and robust standard errors clustered at the community level. As pre-registered, we present findings using both an ordinal and dichotomous measure of vaccine acceptance.

As Fig. 4 shows, all endorsements failed to produce substantive or statistical differences in vaccine acceptance (both measures) in the first survey. As discussed in our comparative analysis section above, overall vaccine hesitancy in our sample was very low, which may have made positive endorsement results less likely due to possible ceiling effects on receptivity. We also speculate—as we did with regard to vaccine receptivity predictors displayed in Fig. 3—that the unavailability of COVID-19 vaccines at the time of fielding the first survey may have led participants to not engage meaningfully with the endorsements because the choice of vaccination was still purely hypothetical. Such a tendency may have attenuated reactions to the endorsements.

In contrast to the first survey, we observe that our endorsement experiment did produce some substantial changes in vaccine acceptance in our second survey. First, relative to baseline levels, COVID-19 vaccine endorsements by Al-Shabaab reduced vaccine acceptance considerably (ordinal: $\hat{\beta } = -0.27$; *p* = 0.004; Cohen’s D = $-0.49$; 95% CI [$-0.46, -0.08$]; dichotomous: $\hat{\beta } = -0.10$; $p =$ 0.023; 95% CI [$-0.19, -0.01$]). These are substantively important effects, as the share of participants agreeing that everyone should be vaccinated falls from 95% to 85% when the statement is accompanied by an Al-Shabaab endorsement.

Second, a vaccine endorsement by customary authorities (clan elders and religious leaders) reduced the share of respondents supporting vaccination efforts by almost 8% points relative to the baseline ($\hat{\beta } = -0.078$; *p* = 0.024; 95% CI [$-0.15, -0.01$]). The estimated effect using the ordinal acceptance measure, however, yields insignificant results ($\hat{\beta } = -0.07$; *p* = 0.20; Cohen’s D = $-0.14$; 95% CI [$-0.18, 0.04$]). Taken together, these findings suggest at the very least that endorsements by customary authorities are unlikely to increase vaccine acceptance and uptake. Indeed, our results indicate that such endorsements are more likely to *reduce* vaccine acceptance in the Somali case, contrary to recent work and policy reports arguing that involving religious leaders may be the most efficient way to increase vaccine demand, particularly in—though not limited to—low-trust environments^3, 12–15, 27, 28^. Consistent with our finding that overall vaccine demand in Somalia is quite high despite low interpersonal trust, we observe little evidence that reported interpersonal or government trust interacts meaningfully with our endorsement treatments (see SI Sect. H). Importantly, given recommendations for endorsements to come from trusted non-state authorities in these contexts, respondents indicated high degrees of *overall* trust toward customary authorities.

Third, we find that a COVID-19 vaccine endorsement by the Ministry of Health had a small positive effect on vaccine acceptance levels ($\hat{\beta }$ = 0.11; *p* = 0.034; Cohen’s D = 0.21; 95% CI [0.01, 0.22]). However, the endorsement did not increase our dichotomous measure of the share of participants agreeing that “everybody should get vaccinated” ($\hat{\beta }$ = 0.01; *p* = 0.59; 95% CI [$-0.04, 0.07$]). Hence, while the government endorsement did not persuade participants to agree with the statement, it may have cemented vaccination acceptance among those who already display favorable attitudes toward vaccination. Fourth and final, while trust in the UN correlates with vaccine receptivity as shown above, we find no endorsement effect of international organizations—which includes the UN as a named organization (see “Methods” section). This indicates that trust in the UN as an institution is not a critical factor in driving support for vaccines, but rather that it likely proxies for trust in the intentions of Western vaccine providers, as noted in the “Discussion” section and consistent with our pre-registration.

### Correlates of vaccine take-up

Next, we examine the predictors of reported vaccination status. To do so, we leverage the panel structure of our data by using covariates from the second survey to predict vaccination status as reported approximately eight months later in the third survey. As mentioned above, vaccination rates were relatively low in our sample ($\approx$ 50% were entirely unvaccinated). In the following, we collapse vaccination status into a binary indicator of whether the respondents had received any vaccination or not (0 = not vaccinated, 1 = vaccinated with at least one dose). 435 (49.94%) of survey 3 respondents who provided their vaccination status reported that they were vaccinated with at least one dose. Figure 5 displays the estimated correlations between individual- and community-level covariates (survey 2) and vaccination status (survey 3). The estimates are based on OLS regressions and include city fixed effects. We cluster standard errors at the community level.

As shown in Fig. 5, most of the predictors of vaccine acceptance in the second survey are also significantly associated with vaccine take-up in the third survey. Participants who relayed a greater negative impact of COVID-19 in the second survey were also more likely to be vaccinated eight months later. People who reported more family sickness and COVID-19 related deaths in the community were more likely to be vaccinated ($\hat{\beta } =$ 0.15; $p =$ 0.002; 95% CI [0.06, 0.23]; and $\hat{\beta } =$ 0.13; $p =$ 0.014; 95% CI [0.03, 0.23], respectively). Economic disruption in the second survey significantly predicts vaccination in the third survey ($\hat{\beta } =$ 0.09; $p =$ 0.015; 95% CI [0.02, 0.17]). Expressing trust in the UN is positively associated with vaccine take-up ($\hat{\beta } =$ 0.14; $p =$ 0.001; 95% CI [0.07, 0.22]), whereas distrust towards the West is negatively, but insignificantly, associated with vaccine take-up ($\hat{\beta } = -0.14$; $p =$ 0.14; 95% CI [$-0.33, 0.05$]). Whereas participants in West-facing occupations did not express greater vaccine acceptance in the second survey (see Fig. 3), they were more likely to have been vaccinated with at least one dose in the third survey ($\hat{\beta } =$ 0.24; *p* = 0.08; 95% CI [$-0.03, 0.52$]). Again, however, this association is insignificant at the conventional 5% level. Further, note that this association is rather imprecisely estimated (SE $=$ 0.13). Receiving community aid was positively associated with vaccine acceptance in the second survey (Fig. 3). Yet, people who lived in communities that received more aid were not more likely to be vaccinated in the third survey ($\hat{\beta } =$ 0.04; $p =$ 0.18; 95% CI [$-0.02, 0.10$]). Finally, greater levels of gender-based violence in the community are positively associated with vaccine take-up ($\hat{\beta } =$ 0.19; $p<$ 0.001; 95% CI [0.10, 0.27]), whereas the individual-level measure of exposure to gender-based violence is not significantly associated with vaccination in the third survey. Being a victim of violence of any type, however, is positively associated with vaccine take-up ($\hat{\beta } =$ 0.19; $p =$ 0.03; 95% CI [0.02, 0.35]).As pre-registered, we also examine the correlations between within-respondent changes in predictors (between rounds 2 and 3) and vaccine take-up (see SI Sect. I).

## Discussion

Understanding the levels, correlates, and possible drivers of vaccine receptivity in conflict regions could prove critical both to informing the response to the current pandemic and to preventing future disease outbreaks. Results from a panel survey of respondents in one of the most conflict-ridden regions of the world, South Central Somalia, offer a few important insights. First, our findings suggest that limited vaccine supply, rather than demand, is more likely to act as a binding constraint on the success of vaccination roll-out efforts. Demand for vaccinations among our Somali sample – as in many other poor regions experiencing conflict (see Fig. 1)—is quite high (around 90%) and outpaces the percentage of respondents reporting having been vaccinated (approximately 50%). High demand is all the more striking given the low interpersonal trust and substantial amount of misinformation about vaccines in circulation among the same sample of respondents (see Fig. 2). Given its high level of conflict, low trust, and high amount of misinformation in circulation, Somalia might be considered a particularly “unlikely” case to observe such high demand should there exist a negative real-world correlation between conflict levels and vaccine receptivity. Consistently, results from our comparative analysis (see Fig. 1 and SI Sect. B) point to wide variation in vaccine receptivity among high-conflict regions: ranging from, for example, 30% in Russia to 76% in Pakistan. In sum, then, a country’s level of conflict does not appear to be a strong predictor of its vaccine receptivity, indicating that, at least at present, fears about low vaccine demand in conflict-ridden countries may be overstated.

Our second set of findings concern the correlates of vaccine receptivity in our study context. We observe that victims of gender-based and other types of violence (and who report more GBV at the community level) are substantially more receptive to vaccination, as are those who report higher levels of COVID-19 related illnesses, deaths, and economic disruption, personally and in their communities. We also find that those who trust the UN and who report having received more aid are more receptive, and those who distrust the West less receptive, to vaccination. Lastly, we observe some evidence that more religious individuals might be more receptive to vaccination.

What might explain these patterns? A number of our findings are consistent with our pre-registered expectations. First, we had anticipated that individuals who had more (positive) exposure to the West and who expressed greater trust in Western-affiliated international organizations would be more receptive to a vaccine produced in, distributed by, and broadly associated with, that region. Results support this notion and underline the importance of trust in vaccine producers for vaccine demand, even if interpersonal and government trust appear to play a smaller role in our sample than in some other contexts^3, 8^. Second, we expected that individuals who experienced greater hardship due to COVID-19—whether it be economic or health-related, or experienced personally or in their communities—would be more receptive to receiving the vaccine, as they perceived comparatively higher costs to not being vaccinated. We find strong support for this expectation; findings mirror results in other contexts showing higher vaccine receptivity among those who, for instance, know someone who fell ill with COVID-19^29^. Note, however, that findings are mixed in this regard: as reported in one study, “People who reported COVID-19 sickness in themselves or family members were no more likely to respond positively to the vaccine question”^22^. Third, we predicted that those who reported greater exposure to gender-based violence during the COVID-19 period would be more open to vaccination, to the extent that they perceived changes due to COVID-19 such as restrictions on mobility—or, more generally, higher economic stress—as to some degree responsible for their increased exposure. We find support for this relationship in the data.

A few of the observed relationships were unanticipated. Notably, we had predicted that those with higher exposure to general (not limited to gender-based) violence would be less receptive to vaccines, both because they viewed falling ill from COVID-19 as a comparatively smaller risk versus other present threats and because they might fear repercussions from groups opposed to vaccination. We also expected that more religious individuals would be less open to vaccination, because they perceived falling ill as comparatively less of a risk and because trusted elites from whom they received information might be less likely to promote vaccination. We observe the opposite relationship. However, we only have a measure of religiosity in survey 1, which as discussed above we view as less reliable as the vaccine was unavailable and thus solely hypothetical to respondents at the time of the survey. Similarly, as regards general violence, we observe the opposite relationship from what we had expected: victims of violence are more, not less, receptive to vaccination. We can only speculate as to the explanation for these more unexpected findings. The finding that receptivity is higher among individuals with greater exposure to violence is consistent with cross-country patterns detailed above indicating that, at the very least, vaccine demand does not appear to be negatively correlated with conflict as many have predicted. It is possible that individuals attribute an increase in violence to COVID-19 and are eager to combat the disease, or else that exposure to violence increases receptivity through other channels—for instance, by increasing individuals’ risk aversion or prosociality^30, 31^. In SI Sect. G, we explore whether exposure to violence and views on the political necessity of violence condition responses to vaccine endorsement by extremist militant group Al-Shabaab. We find some support for the notion that those with greater exposure to violence, and who are more accepting of violence as a political necessity, respond less negatively to an Al-Shabaab endorsement. These findings call for future research into the relationship between exposure to violence and responses to public health messaging by extremist militant groups, and into the mechanisms connecting violence to vaccination receptivity.

Our third set of findings concern efforts to increase vaccine receptivity through elite endorsements. We observe that endorsements from Al-Shabaab have a strong *negative* effect on receptivity; we also find some evidence of a negative effect of endorsements from customary leaders (clan and religious elders), as well as of a positive effect of endorsements from government health authorities. The latter, positive endorsement effect is consistent with evidence from other contexts indicating that in the aggregate, individuals follow COVID-19 vaccine advice from authorities^32^. The negative endorsement effect of customary leaders and of Al-Shabaab, on the other hand, stands in contrast to policy recommendations that endorsements from non-state elites should be pursued in low-trust, conflict-ridden regions, as well as to findings from other contexts that such endorsements can be effective^3, 12, 13, 15, 27, 28^.

What explains our endorsement findings? Drawing on research on source credibility and the theoretical framework of Lupia^33^, we interpret the negative endorsement effects of customary authorities and Al-Shabaab as being related to respondents’ negative perceptions of these actors’ relative expertise and commonality of interests as regards vaccine advice. Indeed, respondents expressed high levels of *overall* trust in customary authorities, with approximately 90% saying that they trusted both clan and religious elders “a great deal” or “a lot”. This indicates that a negative endorsement effect from these sources is not attributable to general distrust of customary authorities but rather a conclusion that they are not well-equipped to provide advice in the specific domain of vaccination. While we did not elicit trust toward Al-Shaabab, research consistently indicates that individuals in Somalia are able to discern Al-Shabaab’s strengths and weaknesses, and relative expertise, in different areas of service provision and make domain-specific responses (either positive or negative) to Al-Shabaab associations accordingly^34^. Regarding relative expertise, we expect that neither actor will be seen as an expert on public health and thus an actor whose advice on the matter carries great weight or meaning. Consistently, scholars find in a study of low- and middle-income countries that health workers, rather than religious leaders or celebrities, are widely viewed as offering the most trustworthy advice as regards obtaining a COVID-19 vaccination^7^. Regarding commonality of interests, we anticipate that actors might see their interests as being at odds with both sets of actors—in particular, with Al-Shabaab, which has committed atrocities against many civilians in the region—and thus might view their endorsement of the vaccine as a sign that the vaccine would, to the contrary, not serve the respondent’s interests. Note, however, that although our sample is far-reaching in the context of South Central Somalia and we took great effort to ensure a relatively representative sample (see SI Sect. K.3), we were nevertheless unable to survey respondents in Al-Shabaab controlled and more rural areas due to safety and logistical concerns. One might imagine that in these regions, individuals might be less willing to express a negative response to an Al-Shabaab endorsed policy or might view them as a comparatively more legitimate authority. Similarly, we can only speculate as to whether our other findings would generalize to such regions. For example, while we would expect predictors to be similarly relevant, one expectation is that overall vaccine take-up would be lower in these areas, due to lesser access and information. We might further imagine that Al-Shabaab’s flip-flopping on the issue of COVID-19 and vaccination—from first claiming that the disease constituted a divine punishment to later going so far as to set up COVID-19 treatment centers—further undercut their perceived credibility on the issue^35, 36^. Whatever the reason, our findings call for careful consideration in the selection of elites to offer vaccine endorsements, as well as for further research on the effectiveness of non-state authority endorsements across different contexts. In the case of vaccine endorsers, more may not always be better.

A fourth set of findings concerns the timing of our surveys. Specifically, our results indicate that predictors of receptivity toward vaccines only crystallized once the possibility of obtaining a vaccine was more than solely a hypothetical one. Predictors in survey 2 not only exhibit strong correlations with concurrent vaccine receptivity, but further predict actual reported vaccine status approximately eight months later in survey 3. We also observe strong endorsement effects. Prior to this stage, in survey 1, we observe weaker relationships of correlates to vaccine receptivity, as well as weaker endorsement effects. These findings underline the importance of considering the timing at which individuals are asked about their vaccine receptivity; results are in line with other studies indicating within-respondent over-time changes in receptivity as well as possible differences due to whether questions were asked when a vaccine was available or not^25, 26^. Recall also that findings are robust to limiting over-time comparisons to the same set of respondents; thus, changes in sample compositions are not responsible for differences in results across survey waves. Results suggest that survey findings may be more reliable once vaccines are available, both because the decision might be more salient at that time and because individuals may be better informed about the relevant disease and the costs and benefits of obtaining a vaccination.

## Methods

### Data

#### Data collection

Panel data was collected in collaboration with the United Nations Development Programme Rule of Law Somalia Office (UNDP RoL Somalia). We collected our data in three waves by inviting a randomly chosen subset of respondents to our first survey to participate in our second and third surveys. Due to the ongoing COVID-19 pandemic, we collected data using phones. To do so, we first obtained a sample of phone numbers using a multi-stage stratified sampling procedure. Second, we completed surveys by phone with individuals who had provided their phone numbers and informed consent to participate and whose identity we were able to verify.

**Figure 6:**
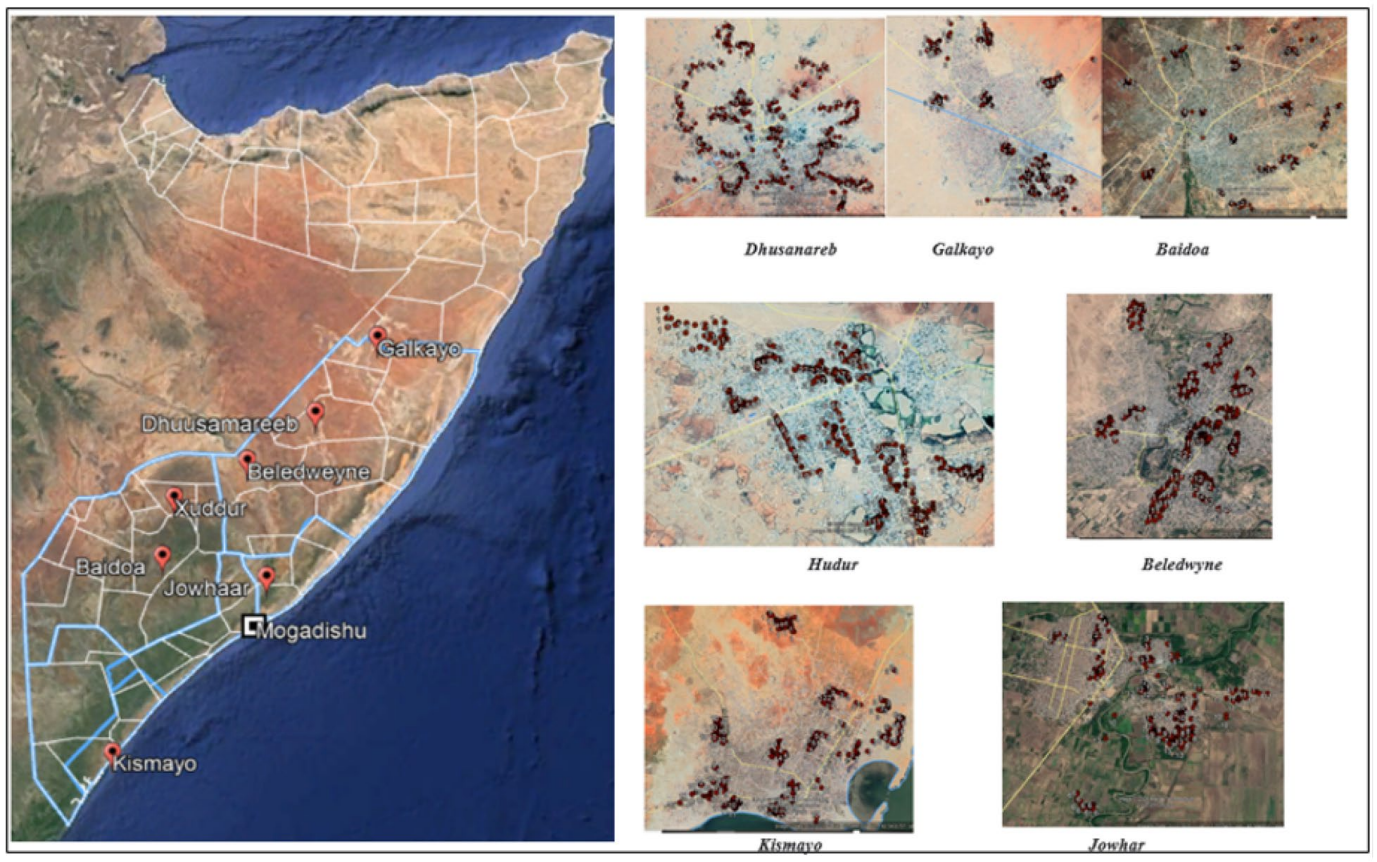
Sampled households. Left map of Somalia showing the location of the seven cities where the survey took place. The seven cities are shown on the right where the red dots are the location of the households who provided consent to participate in the survey. The red dots are clustered around separate communities in each of the seven cities. Maps were generated using Google Earth Pro (open access). Individual cluster/neighborhood names and GPS locations cannot be shared.

To collect phone numbers, we worked with UNDP Somalia and the Ministry of Justice to carry out a listing exercise. Our primary sampling units comprised seven cities in Somalia from five Federal Member States: Kismayo, Hudur, Baidoa, Galkayo, Beledwyne, Dhusanareb, and Jowhaar. Our secondary sampling units consisted of communities/neighborhoods in each city; we randomly sampled a total of 83 across the seven cities, 12 in each except for Hudur (13) and Jowhaar and Galkayo (both 11) due the make up of these cities. As our endorsement experiment was randomized at the individual level, all treatment conditions were represented within each community/neighborhood cluster. We thus far exceed the rule-of-thumb of 40 clusters per treatment arm for our endorsement experiment^37^. To obtain our tertiary sampling units, we randomly sampled approximately 20 households from each community/neighborhood cluster. We sampled households using a “random walk” method: we pre-selected a starting point in each cluster, randomly chose a direction to walk, and asked enumerators to select every third household when walking in that specific direction. See Fig. 6 for the geo-coded location of sampled households across our seven cities.

Individuals from these households—stratified by age and gender using a selection table—make up our ultimate sampling units. From sampled households, enumerators randomly chose a person above 18 years of age and elicited their informed oral consent to participate in a survey. After consenting, potential survey respondents were asked to provide up to two phone numbers at which they could be contacted. Due to the ongoing COVID-19 pandemic, we ensured that enumerators adhered to safety guidelines during this elicitation: they kept five feet of distance between themselves and a respondent, wore personal protective equipment (PPE) gear in the form of facemasks and gloves, and made sure to spend no more than one minute collecting phone numbers. Respondents who provided their consent were told that they would be called to participate in the first and possible subsequent surveys. Our listing exercise was completed between May and June 2020 and resulted in a final sample of approximately 3000 phone numbers.

One of the authors on this paper trained the seven enumerators and the team leader in sampling and survey methodology. Surveys were programmed using the World Bank software Survey Solutions and were conducted in Somali. Before collecting data, we fielded a pre-test of the survey to check for language comprehension, question sensitivity, survey flow, length of interview, and data quality. GPS data was collected to ensure that sampling was implemented according to our design. Data was downloaded from the server at the end of each working day and checked for quality control by one of the authors on this paper. Feedback was then communicated via the team leader to each enumerator each day. All data were stored confidentially in password protected files with access only by the UNDP Somalia Office. We ensured participants’ anonymity and at no point collected information on their names; our publicly-available dataset will not include potentially identifying information such as phone numbers or household GPS coordinates. Because we were not permitted to collect individuals’ names, when we called an individual’s provided phone number from round 1 it is possible that a different individual answered the phone in rounds 2 or 3. To increase our confidence that the same person answered a given phone number, when conducting within-respondent analyses, we only consider a respondent as the same across waves if they used the same phone number and provided the same gender and age (within five years). Our study received Institutional Review Board approval from the University of Essex (Reference #ETH1920-1801), and respondents were debriefed as to the study’s purpose at the conclusion of the survey. Reassuringly, we do not observe any long-term effects of our endorsements; thus for instance, while the Al-Shabaab endorsement results in lesser receptivity in survey 2, we do not find that those who received the endorsement in survey 2 were less likely to report being vaccinated in survey three (see SI Sect. J). All research was performed in accordance with relevant guidelines and regulations, and informed consent was obtained from all participants.

Data for round 1 (Survey 1) of our survey was collected from January 30, 2021, to February 27, 2021. Enumerators were trained beforehand and were given a three-day refresher session in July 2021 by the team leader prior to the second round of data collection (Survey 2), which was completed between August 3, 2021, and August 17, 2021. We conducted our final round of data collection (Survey 3) between March 17, 2022 and April 2, 2022. Survey 1 contains 1672 responses, which corresponds closely to our aim of 20 respondents in each of the 83 sampled communities. We sampled the maximally feasible number of respondents within some important logistical constraints related to collecting survey data in Somalia as well as subject to budgetary constraints of our implementing partners in the UN and Ministry of Justice. Our pre-registration also reports results from a minimum detectable effect size analysis which suggests that we were sufficiently powered to detect relatively small effect sizes (around Cohen’s *D*=0.25). We randomly re-contacted approximately half of our Survey 1 sample in each sampled community to complete Surveys 2 ($N=880$) and 3 ($N=908$). Enumerators were instructed to call multiple times if they were unsuccessful the first time; through this method, we ensured almost no attrition between survey waves. For round 3, we have 908 respondents interviewed from 74 communities from the same seven cities. These 908 respondents were first selected from the 880 respondents of Round 2 and then the remainder from Round 1. Though the initial plan of UNDP Somalia was to interview all subjects interviewed in Round 1, due to budget constraints, we only managed this smaller sub-sample ($N=908$). Our samples are generally balanced across survey waves, although we obtained a larger proportion of women in Survey 1 due to their greater availability when contacted. Results are robust to limiting cross-wave analyses to the same set of individuals and to the inclusion of demographic controls. Our final sample for the three rounds from each of the seven cities are as: Kismayo (239/122/115), Jowar (220/120/113), Baidoa (220/130/109), Belet Weyne (242/130/152), Dhusanareb (241/130/157), Hudur (261/138/158) and Galkayo (230/110/104). Appendix Sect. K contains more information on our sampling design.

Our comparative analysis presented in Fig. 1 additionally relies on a few publicly available datasets^7, 22–24^. See SI Table A1 for more information.

#### Dependent variables

The main outcome of interest in our panel survey is vaccine acceptance. In the first and second rounds of data collection, we asked respondents how much they agreed with the statement that “everyone should be vaccinated”. Responses were elicited on a four-point scale ranging from “strongly disagree” (1) to “strongly agree” (4), which we also collapse into a binary measure of vaccine acceptance (“agree” or “strongly agree”, equal to 1) or not (“disagree” or “strongly disagree”, equal to 0). Lastly, respondents in the third survey round were asked: “Did you take the COVID-19 vaccine?” Options included “no”, “yes, but only one dose”, “yes–two doses”, or “yes–three doses (two $+$ booster dose)”. We consider a binary variable equal to one if a respondent reported receiving at least one dose and zero if they reported no doses. All three questions on vaccine acceptance also included “don’t know” and “refuse to answer” options, which we recode in our analysis to missing. Measures of vaccine acceptance used in our comparative analysis varied within and across studies; precise question wording can be found in SI Table A1.

#### Explanatory variables

In survey waves 1 and 2 of our panel, questions on vaccine acceptance were preceded by endorsements from a randomly assigned source (or no source for subjects randomly assigned to our baseline condition). In survey round 2, a programming error resulted in 83 respondents being assigned to both the baseline and Al-Shabaab endorsement conditions, and in 88 to no condition. The former respondents first answered the baseline vaccine receptivity question prior to being asked the question including an Al-Shabaab endorsement. We accordingly treat these respondents as being assigned to the baseline condition and use their answer to that question in our main analysis; however, as we demonstrate in SI Sect. C, results are robust to the exclusion of these respondents and to a number of other tests. The latter respondents did not answer a question on vaccine receptivity and are accordingly dropped from all such analyses. Depending on one’s treatment assignment, survey 1 respondents were asked: “When the COVID-19 vaccine is available in Somalia, [(*no text, Baseline Condition*)/The Federal Government of Somalia (FGS)/The Traditional Authorities (clan elders & religious leaders) in your community/the World Health Organization (UN health organization)/The African Union believe(s) that] everyone should be vaccinated. Do you agree with this statement?” Survey 2 included slightly modified groups and question wording: “Now that the COVID-19 vaccine is available in your community, [(*no text, Baseline Condition*)/the Ministry of Health-Somalia/The Traditional Authorities (clan elders and religious leaders) in your community/the International Communities including the United Nations and the African Union/the Armed Group Al-Shabaab believe(s) that] everyone should be vaccinated. Do you agree with this statement that everyone should be vaccinated?” We made three changes to our treatments between surveys 1 and 2. First, we grouped the WHO and the African Union (AU) together in survey 2 because the WHO collaborated with AMISOM (AU) to deliver COVID-19 vaccines. Hence, we expected that these two actors would be seen as one. Second, we included Al-Shabaab as an endorser because of a change in Al-Shabaab’s strategy, which made them a valid endorser of COVID-19 vaccination at the time of survey 2 (but not survey 1). Third, we changed the government authority endorsing vaccination to, more specifically, the Ministry of Health-Somalia, which we expected would be viewed as having relatively high expertise on the issue. To evaluate endorsement treatment effects in these survey waves, we compare vaccine acceptance among individuals randomly assigned to our baseline group (0) versus each of our different treatment conditions (1).

We also pre-registered as explanatory variables a number of survey questions that we expected would be correlated with vaccine receptivity. We measure whether an individual reported being or a member of their household being a *victim of violence* in the previous 12 months (survey 1) or in the six months since the previous survey (survey 2), which we recode as either no violence (0) or violence on one or more occasion (1); *victim of gender-based violence (GBV)*, which refers to whether an individual reported in a follow-up question that the violence was against women (1) or whether they did not report any violence (0); *GBV in the community*, which asked respondents in survey 2 how much they agreed (on a 1-4 scale) with the statement that, “Since the last six months (after we interviewed you in February), there has been an increase in violence against women in my community”; *sickness in their family*, which asked respondents to share how many members of their family had been sick with COVID-19 (e.g., cough, throat fever, chest pain or shortness of breath) and was coded as either “none” (1), “1–2 members” (2), “3–5 members” (3), or “more than 5 members” (4); *deaths* and *sickness in the community*, which used the same phrasing and response categories as *sickness in their family* to measure estimated deaths and sickness, respectively, due to COVID-19 at the community level; *economic disruption*, which asked respondents to estimate the percentage decline in household income due to COVID-19 and which was recoded to distinguish between households that made more money during COVID-19 than prior (−1), households that were unaffected (0), and households whose incomes were negatively affected by COVID-19 by an estimated 10% (1), 20% (2), over 50% (3), or over 80% (4); and religiosity, which asked respondents how often they read or listen to the Quran from “very rarely” (1) to “always” (4).

We also sought to measure exposure and attitudes toward the West, which we viewed as potentially relevant to informing receptivity toward vaccines produced in and delivered by the region. *Trust in the UN* measures respondents’ trust in the United Nations and varies from “no trust at all” (1) to “a great deal of trust” (4); *community aid* measures whether a respondent reported that their community had received COVID-19 related aid (1) or not (0); *distrust in the West*, which measures whether a respondent said that Western countries were mainly motivated to give foreign aid to Somalia in order to “gain influence in the country” (1) versus to promote its economic development, internal security and stability, to empower its civil society organizations, or to improve the lives of ordinary citizens (0); and *West-facing occupation*, which measured whether an individual reported being employed in a government or security forces position or with a NGO or INGO (1) or not (0).

Finally, for our comparative analysis, we consider two explanatory variables. First, a country’s pre-pandemic gross national income (GNI)^23^. Second, a country’s score on the Global Peace Index (GPI)^24^. Lower (higher) GPI values correspond to more (less) peace, and for the year and set of countries in our sample ranges from 7 (Canada, most peaceful) to 155 (Russia, least peaceful), with Somalia earning a score of 154. In SI Sect. B, we also explore robustness of results to using alternative, non-ranked measures of exposure to conflict.

### Analysis

In our analysis, we run a series of simple OLS regressions. In these regressions, our dependent variable is either an individual’s vaccine receptivity (surveys 1 and 2, measured on ordinal and binary scales) or reported vaccine status (survey 3, binary scale). Our independent variable is either a dummy for endorsement treatment status, a predictor measured on the scale detailed in the previous subsection (either concurrent or lagged, that is we also evaluate correlations between predictors from survey 2 and reported vaccination status in survey 3), or changes in a predictor variable between surveys 2 and 3. Analyses evaluating how a predictor at time *t* relates to vaccination status at time $t+1$ are necessarily within-subject; other analyses are between-subject, though we explore robustness to limiting comparisons across surveys to the same set of respondents. We cluster standard errors at the community level and include city fixed effects. These different specifications – restricting the sample, including city fixed effects, and within-respondent analyses—all yield similar results, indicating that differences in vaccine receptivity across rounds are not driven by changes in the sample compositions. Lastly, for our comparative analysis, we regress average vaccine receptivity in a given study in a given country on that country’s pre-pandemic GNI or GPI score, and we probe robustness to the inclusion of study and country fixed effects.

### Ethical standards

This study received Institutional Review Board approval from the University of Essex (Reference #ETH1920-1801) and was conducted in line with the ethical standards contained in the 1964 Declaration of Helsinki and its later amendments.

### Pre-registration

The study was pre-registered with the Open Science Framework (https://osf.io/y5tj8/?view_only=ffbad5cf4382486e93de167ec1b2199c).

### Supplementary Information

Supplementary Information.

## Data Availability

The datasets used and/or analysed during the current study are available from the corresponding author on reasonable request.
