# Supplementary material for: Despite misinformation, low trust, and conflict in Somalia, high demand for vaccines and a negative endorsement effect of non-state authorities
Source: Sci Rep. 2023 Dec 7;13:21689. doi: 10.1038/s41598-023-48389-7 (PMC10709303; doi:10.1038/s41598-023-48389-7)
Supplement: Supplementary file 1 — Supplementary Information. [file 41598_2023_48389_MOESM1_ESM.pdf]

Supplementary Information

Despite Misinformation, Low Trust, and Conflict in Somalia, High Demand for Vaccines and a Negative Endorsement Effect of non-State Authorities

Table of Contents

A Study Questionnaires **A2**
B Additional Information: Comparative Studies **A3**
B.1 Alternative Measures for Conflict A4
C Alternative Specifications **A7**
D Regression Tables for Fig. 3-4 . . . . **A9**
E Figure 3 using only respondents from both waves **A11**
F Figure 3 including additional covariates. **A12**
G Conditional Effects of Al-Shabaab Endorsement **A15**
H Conditional Effects of Traditional Authorities Endorsement **A17**
I Estimating Within-Respondent Changes **A19**
J The Effect of Endorsements on Vaccine Take-up **A20**
K Additional Sampling Information **A21**
K.1 Script for Enumerators A21
K.2 Generalizability- Comparing Our Study Sample with the World Bank 2021 Telephone Survey . . A21
K.3 Data Comparison with World Bank Survey A22
L Attrition across demographic groups and survey rounds **A25**
M Anonymized Photographs: Phone Number Collection. **A25**

A Study Questionnaires

To see study questionnaires for each of our three survey waves, follow this link.

B Additional Information: Comparative Studies

Table A1: Vaccine Acceptance, GPI, and GNI Across Countries and Studies (Figure 1)

Country	ISO Code	Acceptance	Source	Data Collection	Measure	GPI Ranking (2019)	GNI ($, 2019)
China	CN	88,62	Lazarus et al. (2021)	June 2020	"If a COVID-19 vaccine is proven safe and effective and is available to me, I will take it"	95	10,310
Brazil	BR	85,36	Lazarus et al. (2021)	June 2020	See above	127	9,220
South Africa	ZA	81,58	Lazarus et al. (2021)	June 2020	See above	132	6,670
South Korea	KR	79,79	Lazarus et al. (2021)	June 2020	See above	52	33,830
Mexico	MX	76,25	Lazarus et al. (2021)	June 2020	See above	138	9,470
United States	US	75,42	Lazarus et al. (2021)	June 2020	See above	125	65,970
India	IN	74,53	Lazarus et al. (2021)	June 2020	See above	142	2,100
Spain	ES	74,33	Lazarus et al. (2021)	June 2020	See above	36	30,380
Ecuador	EC	71,93	Lazarus et al. (2021)	June 2020	See above	78	6,090
United Kingdom	GB	71,48	Lazarus et al. (2021)	June 2020	See above	35	43,460
Italy	IT	70,79	Lazarus et al. (2021)	June 2020	See above	34	34,940
Canada	CA	68,74	Lazarus et al. (2021)	June 2020	See above	7	46,540
Germany	DE	68,42	Lazarus et al. (2021)	June 2020	See above	22	49,190
Singapore	SG	67,94	Lazarus et al. (2021)	June 2020	See above	10	58,060
Sweden	SE	65,23	Lazarus et al. (2021)	June 2020	See above	19	56,420
Nigeria	NG	65,22	Lazarus et al. (2021)	June 2020	See above	149	2,030
France	FR	58,89	Lazarus et al. (2021)	June 2020	See above	59	42,550
Poland	PL	56,31	Lazarus et al. (2021)	June 2020	See above	26	15,360
Russia	RU	54,85	Lazarus et al. (2021)	June 2020	See above	155	11,280
Burkina Faso	BF	66,5	Arce et al. (2021)	Oct-Dec 2020	If a COVID-19 vaccine became available in Burkina Faso, would you take it?	109	770
Colombia	CO	74,9	Arce et al. (2021)	August 2020	If a COVID-19 vaccine became available in Colombia, would you take it?	145	6,570
India	IN	84,3	Arce et al. (2021)	June 2020-Jan 2021	If a vaccine for coronavirus gets introduced, would you like to get it?	142	2,100
Mozambique	MZ	89,1	Arce et al. (2021)	Oct-Nov 2020	When a COVID-19 vaccine becomes available in the future, would you take it?	107	490
Nepal	NP	96,6	Arce et al. (2021)	Dec 2020	Should a vaccine against COVID become available in Nepal, would you take it?	81	1,230
Nigeria	NG	76,2	Arce et al. (2021)	Nov-Dec 2020	If a COVID-19 vaccine became available in Nigeria, would you take it?	149	2,030
Pakistan 1	PK	76,1	Arce et al. (2021)	July-Sept 2020	If a vaccine against the coronavirus becomes available, do you plan to get vaccinated?	152	1,620
Pakistan 2	PK	66,5	Arce et al. (2021)	Sept-Oct 2020	If a vaccine against the coronavirus becomes available, do you plan to get vaccinated?	152	1,620
Rwanda	RW	84,9	Arce et al. (2021)	Oct-Nov 2020	If a COVID-19 vaccine became available in Rwanda, would you take it?	84	830
Sierra Leone 1	SL	78	Arce et al. (2021)	Oct 2020	If a COVID-19 vaccine became available in Sierra Leone, would you take it?	54	530
Sierra Leone 2	SL	87,9	Arce et al. (2021)	Oct 2020-Jan 2021	Should a vaccine against COVID become available in Sierra Leone, would you take it?	54	530
Uganda 1	UG	85,8	Arce et al. (2021)	Sept-Dec 2020	When a COVID-19 vaccine becomes available in Uganda, would you take it?	115	780
Uganda 2	UG	76,5	Arce et al. (2021)	Nov-Dec 2020	If a COVID-19 vaccine becomes available in Uganda, would you take it?	115	780
Russia	RU	30,4	Arce et al. (2021)	Nov-Dec 2020	If a COVID-19 vaccine became available in Russia, would you take it?	155	11,280
United States	US	64,6	Arce et al. (2021)	Dec 2020	If a COVID-19 vaccine becomes available in the United States, would you take it?	125	65,970
Somalia 1	SO	89,8	Aarslew et al. (2022)	Jan-Feb 2021	When the COVID-19 vaccine is available in Somalia, everyone should be vaccinated. Do you agree with this statement?	154	420
Somalia 2	SO	93,75	Aarslew et al. (2022)	Aug 2021	Now that the COVID-19 vaccine is available in your community, everyone should be vaccinated. Do you agree with this statement that everyone should be vaccinated?	154	420

Notes: This table presents data on vaccine acceptance, GNI, and GPI across studies and countries, corresponding to results presented in Figure 1 in the main text. The Global Peace Index, or GPI, is calculated for 163 countries using 23 qualitative and quantitative indicators and is produced by the Institute for Economics & Peace; lower (higher) numbers correspond to more (less) estimated peace in a given country (for more information, see here: `https://www.visionofhumanity.org/maps/#/`). Gross National Income (per capita), or GNI, is sourced from the World Bank, see here: `https://data.worldbank.org/indicator/NY.GNP.PCAP.CD`.

Table A2: Vaccine Acceptance, GNI, and GPI: Regression Results for Figure 1

	(1)	(2)	(3)	(4)	(5)	(6)
Gross National Income (2019)	-0.00*	-0.00	0.00			
	(0.00)	(0.00)	(0.00)			
Global Peace Index (2019)				0.02	-0.03	0.50
				(0.04)	(0.05)	(0.63)
# Countries	36	36	36	36	36	36
Fixed Effects	None	Study	Study & Country	None	Study	Study & Country

Notes: *$p < 0.05$, **$p < 0.01$, ***$p < 0.001$

B.1 Alternative Measures for Conflict

In this Section, we reproduce Figure 1 and Appendix Table A2 using two alternative measures to the Global Peace Index [1] for pre-pandemic exposure to conflict. Specifically, we use a non-ranked based comparative measure of conflict exposure – the estimated number of country-level fatalities in 2019 – from two different datasets: the Uppsala Conflict Data Program (UCDP [2]) and the Armed Conflict Location and Event Data Project (ACLED [3]). We use the UCDP's Georeferenced Event Dataset, which covers organized violence events (categorized either as state-, non-state, or one-sided violence) with at least one fatality worldwide [2]. The ACLED dataset contains a wider range of events: battles, explosions/remote violence, protests, riots, strategic development, violence against civilians, and events with zero fatalities [3].

In each dataset, we take the total number of 2019 fatalities recorded for each country displayed in Figure 1. Results are displayed visually in Figure A1. In Table A3, we present regression output including country and study fixed effects (panel A), as well as only including countries with at least one fatality (panel B), excluding an outlier country (Mexico, panel C), and using an alternative measure for conflict exposure that captures a country's number of recorded violent events as opposed to the number of fatalities (panel D). Results with these alternative measures for conflict exposure are consistent with those using Global Peace Index data [1] and indicate, at least for our sample of studies and countries, that there is no robust association between a country's level of conflict exposure and its vaccine receptivity.

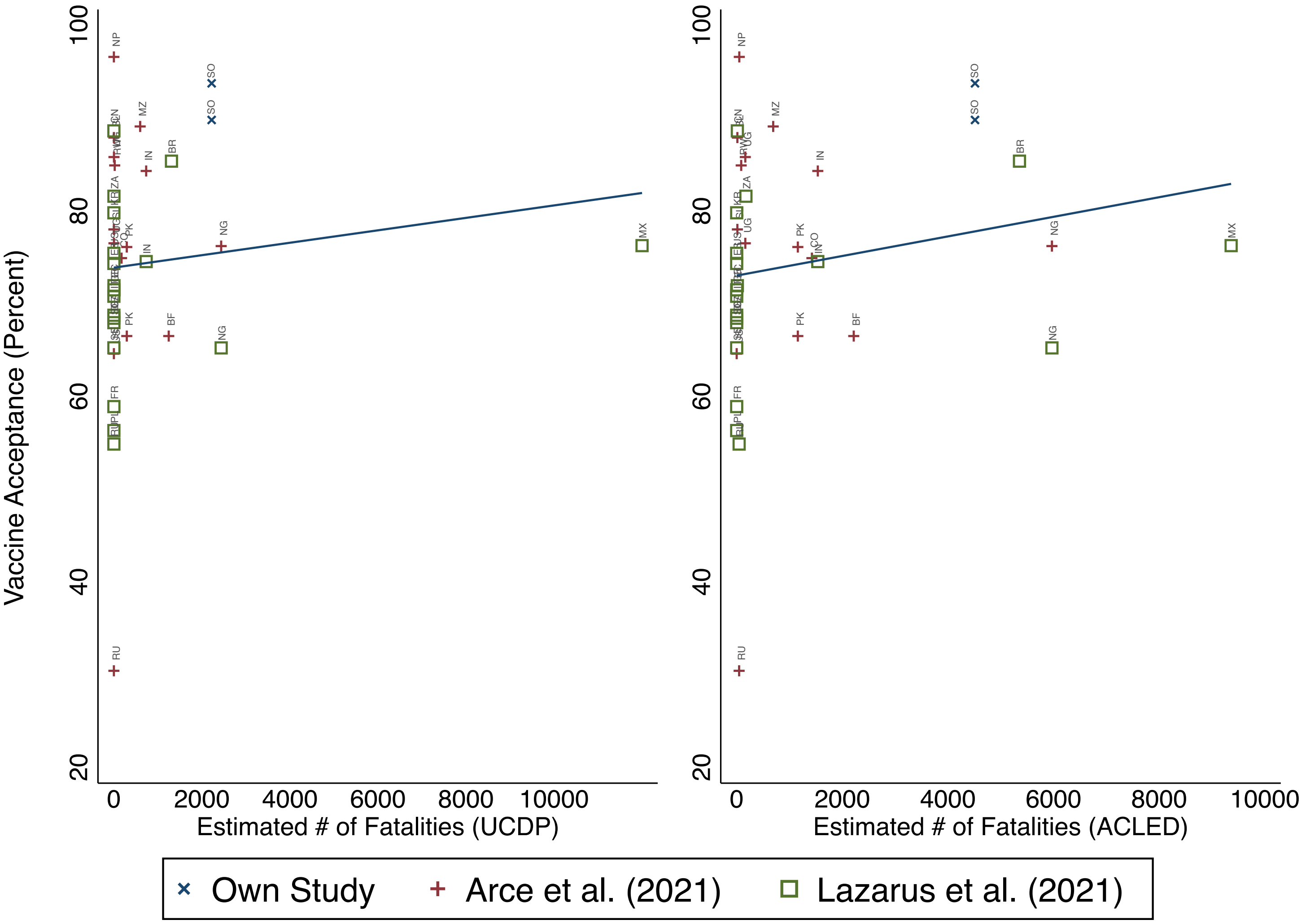

Notes: This figure displays vaccine acceptance across countries; we include our own estimates for Somalia across two waves, as well as estimates reported in additional, global studies [4,5]. We explore relationships between vaccine acceptance and pre-pandemic conflict (using estimates of country-level fatalities in 2019 from either the UCDP dataset [2] in the left panel or the ACLED dataset [3] in the right panel).

Figure A1: Replication of Figure 1 with Alternative Conflict Measures

	UCDP Data			ACLED Data		
	(1)	(2)	(3)	(4)	(5)	(6)
**Panel A: Fatalities, all Countries**						
Fatalities (2019)	0.00	0.00	-0.01	0.00	0.00	-0.01
	(0.00)	(0.00)	(0.01)	(0.00)	(0.00)	(0.01)
N	36	36	36	36	36	36
**Panel B: Fatalities > 0**						
Fatalities (2019)	-0.00	-0.00	-0.02*	0.00	-0.00	-0.01
	(0.00)	(0.00)	(0.01)	(0.00)	(0.00)	(0.01)
N	19	19	19	24	24	24
**Panel C: Fatalities, Excluding Mexico**						
Fatalities (2019)	0.00	0.00	-0.01	0.00	0.00	-0.01
	(0.00)	(0.00)	(0.01)	(0.00)	(0.00)	(0.01)
N	35	35	35	35	35	35
**Panel D: Violent Events, All Countries**						
Violent Events (2019)	0.01	0.01	-0.05	0.00	0.00	0.02
	(0.01)	(0.01)	(0.07)	(0.00)	(0.00)	(0.02)
N	36	36	36	36	36	36
Fixed Effects	None	Study	Study & Country	None	Study	Study & Country

* $p<0.05$, ** $p<0.01$, *** $p<0.001$

Notes: This table displays the relationship between a country's pre-pandemic conflict exposure using data either from UCDP (columns 1-3 [2]) or ACLED (columns 4-6 [3]) and its level of vaccine receptivity as measured through surveys. Panels A-C use the number of fatalities in 2019 as the measure of conflict and explore robustness to only including countries with at least one fatality recorded (panel B) and to excluding an outlier country (Mexico, panel C). Panel D uses the number of violent events recorded in 2019 as the measure of conflict. Columns 2 and 5 display results including survey study fixed effects, and columns 3 and 6 display results including both study and country fixed effects.

Table A3: Regression Results Corresponding to Figure A1

C Alternative Specifications

As noted in the main text, a programming error resulted in some individuals who should have been assigned to the baseline condition instead being assigned to both that condition and the Al-Shabaab condition (with the baseline condition vaccine receptivity question asked first). In our main analysis, we treat these respondents as having been assigned to the baseline condition and we control for duplicate assignment. However, one might be concerned that these individuals were contaminated in some way – for instance, if enumerators asked the baseline question with a different intonation knowing that the Al-Shabaab endorsement was to follow it.

While we find such a possibility unlikely, we address this concern in a few ways below. Reassuringly, we find that our endorsement experiment results are robust to these exercises. First, we observe that results are robust to excluding individuals who were assigned to multiple endorsement conditions from our analysis. Second, we find that results are robust to controlling for observables on which there was imbalance between treatment conditions in survey 2. Third, we find that results are unchanged if we categorize those assigned to multiple endorsement conditions as Al-Shabaab treatment respondents (taking their answer to that question instead), as opposed to baseline respondents. Fourth, we find that results are robust to treating these respondents as baseline respondents but not controlling for duplicated assignment. Fifth, we note that results across surveys 1 and 2 are robust to limiting our comparative analyses to the *same set of respondents* (see Fig. A3), further increasing our confidence that differences are not due to differences in our samples across waves or treatment conditions.

Figure A2: Alternative Specification of Endorsement Experiment: Findings are robust to excluding duplicates, adjusting for imbalances, not controlling for duplication status, and coding duplicate respondents as being in Al-Shabaab condition.

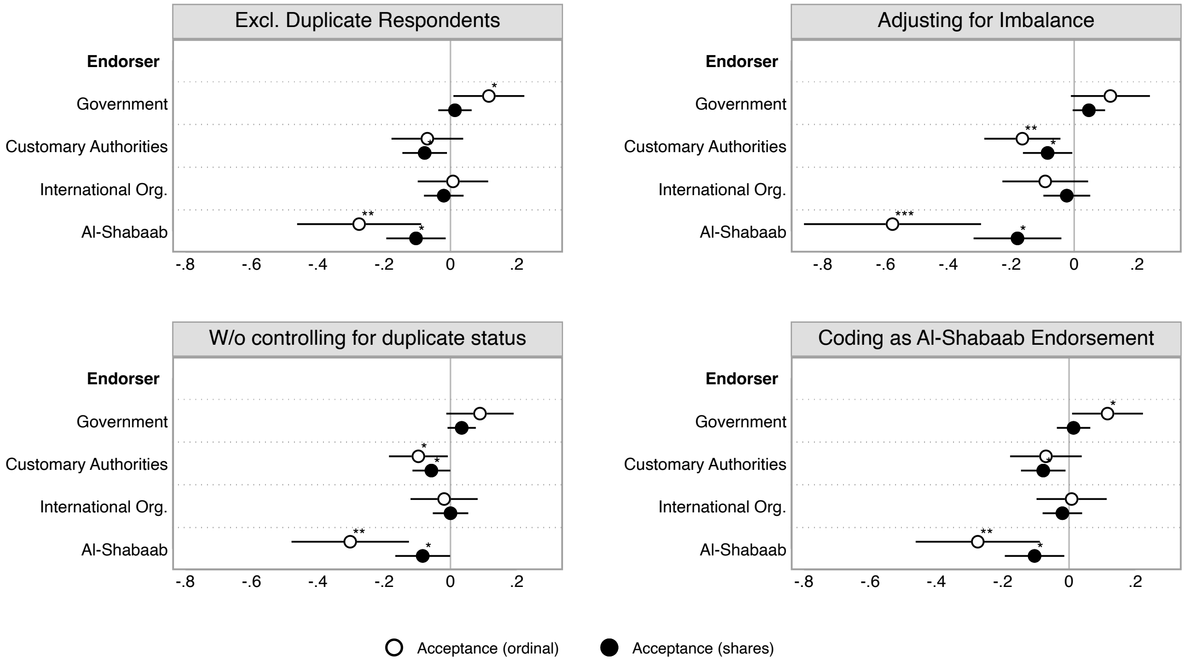

Note: Estimates are based on unstandardized OLS regressions with city fixed effects. Bars are 95% confidence intervals based on robust standard errors clustered on communities. Upper left-hand panel displays findings excluding 89 duplicated respondents, upper right-hand panel displays findings adjusting for gender, age, marital status and the family size (no. of children), lower left-hand panel shows findings without covariate adjusting for duplication status, and the lower right-hand panel shows findings when duplicated respondents are coded as being in the Al-Shabaab endorsement condition. * $p < 0.05$, ** $p < 0.01$, *** $p < 0.001$ (two-sided tests).

D Regression Tables for Fig. 3-4

This section presents regression tables for figures 3 and 4 in the main text. The tables (like the main figures) are presented for survey round 1 and 2 separately.

Table A4: Table for Fig. 3 (S1)

	Acceptance	Acc.	Acc.	Acc.	Acc.	Acc.	Acc.	Acc.	Acc.	Acc.	Acc.
Religiosity	0.12***										
	(0.03)										
Victim (violence)		0.08									
		(0.05)									
Economic disruption			-0.05*								
			(0.02)								
Sickness (community)				0.10***							
				(0.03)							
Deaths (community)					0.01						
					(0.02)						
Distrust West						0.12					
						(0.09)					
Trust (UN)							0.00				
							(0.03)				
Community aid								0.04			
								(0.02)			
West-facing Occ.									0.08		
									(0.06)		
Victim of GBV										0.12	
										(0.09)	
Sickness (family)											-0.06
											(0.03)
Constant	2.29***	2.72***	2.82***	2.37***	2.65***	2.67***	2.69***	2.58***	2.68***	2.80***	2.81***
	(0.15)	(0.15)	(0.15)	(0.17)	(0.17)	(0.15)	(0.17)	(0.15)	(0.15)	(0.15)	(0.18)
adj. R^2	0.070	0.032	0.054	0.060	0.039	0.041	0.037	0.044	0.038	0.012	0.046
N	1649	1588	1645	1632	1580	1647	1638	1649	1649	1352	1649

Note: Unstandardized OLS regression estimates. Robust standard errors clustered on community in parentheses.
* $p < 0.05$, ** $p < 0.01$, *** $p < 0.001$ (two-sided tests).

Table A5: Table for Fig. 4 (S1)

	Acceptance (Shares)	Acceptance (Ordinal)
Govenment	-0.01	0.02
	(0.02)	(0.04)
Trad. Authorities	0.00	0.01
	(0.02)	(0.04)
Int. Org.	0.00	0.03
	(0.02)	(0.04)
Amisom	-0.01	-0.01
	(0.02)	(0.05)
Constant	0.75***	2.69***
	(0.07)	(0.16)
adj. R^2	0.049	0.038
N	1649	1649

Note: Unstandardized OLS regression estimates. Robust standard errors clustered on community in parentheses.
* $p < 0.05$, ** $p < 0.01$, *** $p < 0.001$ (two-sided tests).

Table A6: Table for Fig. 3 (S2)

	Acceptance	Acc.	Acc.	Acc.	Acc.	Acc.	Acc.	Acc.	Acc.	Acc.
Victim (violence)	0.20**									
	(0.07)									
Victim of GBV		0.32***								
		(0.08)								
Sickness (family)			0.18***							
			(0.03)							
Deaths (community)				0.13***						
				(0.03)						
Economic disruption					0.10***					
					(0.02)					
Trust (UN)						0.16***				
						(0.05)				
Community aid							0.13***			
							(0.02)			
Distrust West								0.12		
								(0.09)		
West-facing Occ.									-0.12	
									(0.08)	
GBV (Community)										0.15**
										(0.05)
Controls										
Experimental Condition	✓	✓	✓	✓	✓	✓	✓	✓	✓	✓
City Fixed Effects	✓	✓	✓	✓	✓	✓	✓	✓	✓	✓
Constant	2.82***	2.84***	2.48***	2.55***	2.62***	2.41***	2.55***	2.67***	2.95***	2.58***
	(0.08)	(0.07)	(0.10)	(0.10)	(0.10)	(0.19)	(0.10)	(0.15)	(0.08)	(0.16)
adj. R^2	0.102	0.138	0.135	0.110	0.115	0.109	0.152	0.039	0.076	0.091
N	757	575	791	777	791	789	792	1656	792	786

Note: Unstandardized OLS regression estimates. Robust standard errors clustered on community in parentheses.
* $p < 0.05$, ** $p < 0.01$, *** $p < 0.001$ (two-sided tests).

Table A7: Table for Fig. 4 (S2)

	Acceptance (Shares)	Acceptance (Ordinal)
Govenment	0.01	0.12*
	(0.03)	(0.05)
Costumary Auth.	-0.08*	-0.07
	(0.03)	(0.05)
Int. Org.	-0.02	0.01
	(0.03)	(0.05)
Al-Shabaab	-0.10*	-0.28**
	(0.05)	(0.09)
Duplicate	-0.04	0.06
	(0.04)	(0.07)
Constant	0.88***	2.98***
	(0.04)	(0.08)
adj. R^2	0.034	0.048
N	792	792

Note: Unstandardized OLS regression estimates. Robust standard errors clustered on community in parentheses.
* $p < 0.05$, ** $p < 0.01$, *** $p < 0.001$ (two-sided tests).

E Figure 3 using only respondents from both waves

This section presents the findings in fig. 3 in the main text with a restricted sample, including only respondents who were sampled in both survey round 1 and 2. As shown in the right-hand panel (Survey 2), the findings largely remain unchanged.

Figure A3: Covid-19 vaccine receptivity predictors (only respondents from both waves)

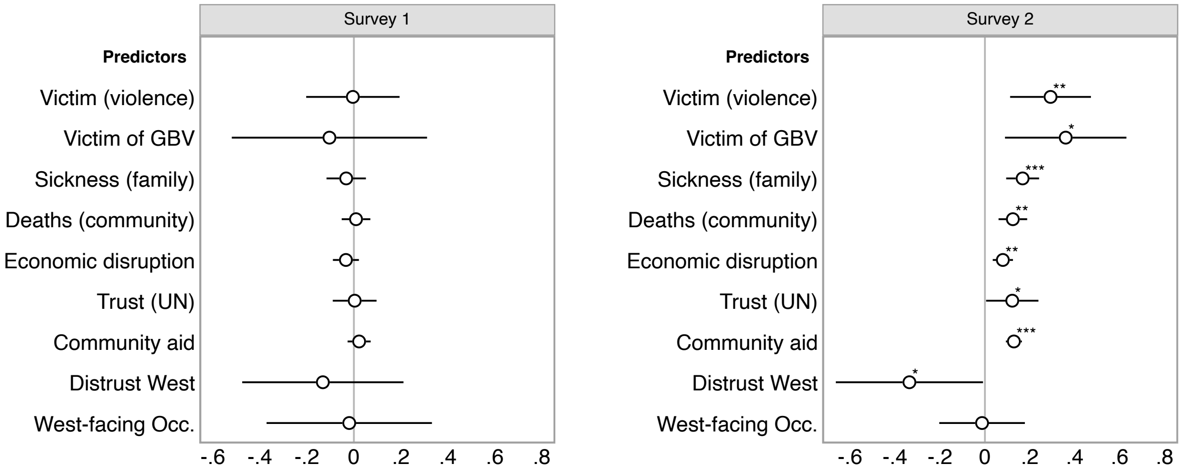

Note: Estimates based on unstandardized OLS regressions with city fixed effects. Whiskers are 95% confidence intervals based on robust standard errors clustered on communities. * $p < 0.05$, ** $p < 0.01$, *** $p < 0.001$ (two-sided tests).

F Figure 3 including additional covariates

The two tables below present results from unstandardized OLS regressions of vaccine receptivity on a series of predictors (similar to fig. 3 in the main text), adjusting for additional covariates. These covariates are age (S1: M = 38.1; SD = 13.61, S2: M = 37.8; SD = 12.85), gender (0 = female, 1 = male, S1: 38.7% male, S2: 50% male), number of children (S1: M = 6.06; SD = 3.14, S2: M = 6.15, SD = 3.49), Education (0 = illiterate, 1 = primary, 2 = secondary, 3 = high school, 4 = university, 5 = only madrassa), clan (0 = Hawiay, 1 = Darood, 2 = Ranweyn or Digil iyo mirifle, 3 = Dir, 4 = other). Fig. A4 depicts the findings. As shown, the findings (see fig. 3 are robust to the inclusion of additional covariate controls. The only difference is that the estimate for distrust in the west turns insignificant ($p = 0.05$). However, the coefficient estimate is similar ($\hat{\beta}$ = -.28) and is rather imprecisely estimated in both models (95% CI [-.57, .0004]).

Figure A4: Covid-19 vaccine receptivity predictors (incl. additional covariates)

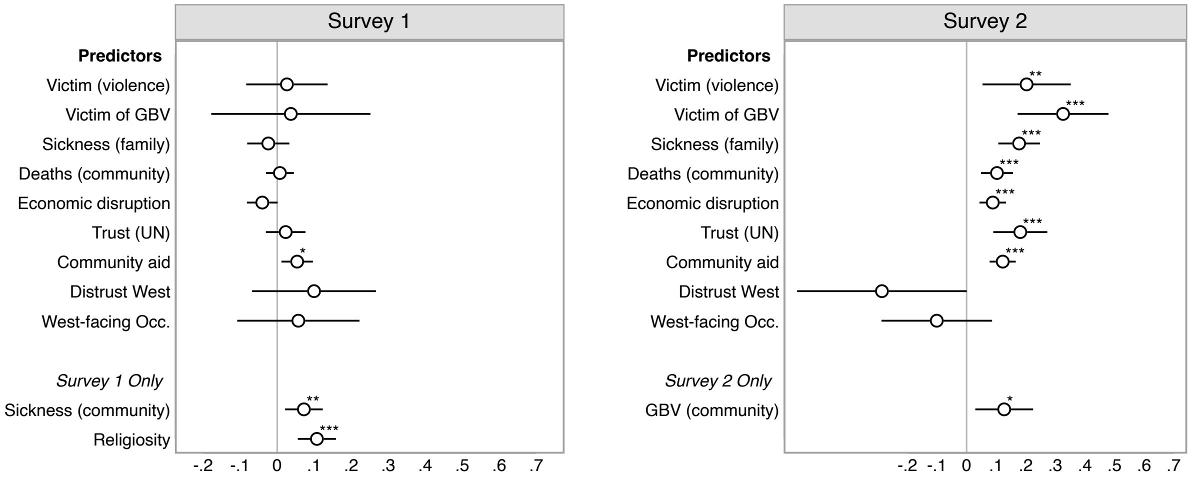

Note: Estimates based on unstandardized OLS regressions with city fixed effects. Controls: treatment assignment, age, gender, education, ethnicity (clan), and family size. Whiskers are 95% confidence intervals based on robust standard errors clustered on communities. * $p < 0.05$, ** $p < 0.01$, *** $p < 0.001$ (two-sided tests).

Table A8: Table for Fig. 3 (S1) [With additional controls]

	Acceptance	Acc.	Acc.	Acc.	Acc.	Acc.	Acc.	Acc.	Acc.	Acc.	Acc.
Religiosity	0.11***										
	(0.03)										
Victim (violence)		0.03									
		(0.06)									
Economic disruption			-0.04								
			(0.02)								
Sickness (community)				0.07**							
				(0.03)							
Deaths (community)					0.01						
					(0.02)						
Distrust West						0.10					
						(0.08)					
Trust (UN)							0.02				
							(0.03)				
Community aid								0.05*			
								(0.02)			
West-facing Occ.									0.06		
									(0.08)		
Victim of GBV										0.04	
										(0.11)	
Sickness (family)											-0.02
											(0.03)
Male	-0.07	-0.07	-0.07	-0.06	-0.07	-0.07	-0.07	-0.07	-0.07	-0.07	-0.07
	(0.04)	(0.04)	(0.04)	(0.04)	(0.04)	(0.04)	(0.04)	(0.04)	(0.04)	(0.04)	(0.04)
Primary	0.01	0.03	0.03	0.01	0.01	0.02	0.01	-0.01	0.02	0.07	0.03
	(0.06)	(0.06)	(0.06)	(0.06)	(0.06)	(0.06)	(0.06)	(0.07)	(0.06)	(0.07)	(0.06)
Secondary	0.06	0.05	0.04	0.04	0.04	0.05	0.04	0.04	0.04	0.11	0.04
	(0.06)	(0.07)	(0.06)	(0.06)	(0.06)	(0.06)	(0.06)	(0.07)	(0.07)	(0.07)	(0.06)
High school	0.09	0.11	0.09	0.11	0.10	0.11	0.11	0.14	0.11	0.18*	0.10
	(0.07)	(0.08)	(0.07)	(0.07)	(0.07)	(0.07)	(0.07)	(0.08)	(0.07)	(0.09)	(0.07)
University	0.21**	0.24**	0.21**	0.23**	0.21**	0.23**	0.23**	0.26**	0.23**	0.25**	0.22**
	(0.08)	(0.08)	(0.07)	(0.08)	(0.08)	(0.08)	(0.07)	(0.08)	(0.08)	(0.09)	(0.08)
Only Madrassa	0.24**	0.25**	0.25***	0.26***	0.24**	0.27***	0.27***	0.29***	0.28***	0.30**	0.26***
	(0.07)	(0.08)	(0.07)	(0.08)	(0.07)	(0.07)	(0.07)	(0.08)	(0.08)	(0.09)	(0.07)
Darood	-0.07	-0.07	-0.08	-0.07	-0.07	-0.07	-0.09	-0.07	-0.08	-0.02	-0.08
	(0.08)	(0.08)	(0.08)	(0.07)	(0.08)	(0.07)	(0.07)	(0.07)	(0.08)	(0.09)	(0.08)
Ranweyn or Digil iyo mirifle	-0.03	-0.03	-0.10	-0.02	-0.00	-0.03	-0.04	-0.01	-0.04	0.02	-0.03
	(0.09)	(0.09)	(0.09)	(0.09)	(0.09)	(0.09)	(0.09)	(0.09)	(0.09)	(0.09)	(0.09)
Dir	0.07	0.09	0.01	0.06	-0.01	0.05	0.10	0.09	0.07	0.25*	0.07
	(0.10)	(0.10)	(0.11)	(0.11)	(0.08)	(0.10)	(0.12)	(0.11)	(0.11)	(0.10)	(0.10)
Other	0.06	0.00	-0.01	0.01	0.03	0.00	0.01	0.06	0.01	0.04	0.01
	(0.10)	(0.12)	(0.12)	(0.11)	(0.11)	(0.12)	(0.11)	(0.12)	(0.12)	(0.13)	(0.12)
No. Children	-0.00	-0.01	-0.01	-0.00	-0.01	-0.01	-0.01	-0.00	-0.01	-0.00	-0.01
	(0.01)	(0.01)	(0.01)	(0.01)	(0.01)	(0.01)	(0.01)	(0.01)	(0.01)	(0.01)	(0.01)
Age	0.00	0.00	0.00	0.00	0.00	0.00	0.00	0.00	0.00	0.00	0.00
	(0.00)	(0.00)	(0.00)	(0.00)	(0.00)	(0.00)	(0.00)	(0.00)	(0.00)	(0.00)	(0.00)
Constant	2.31***	2.66***	2.77***	2.40***	2.62***	2.63***	2.60***	2.48***	2.64***	2.70***	2.71***
	(0.16)	(0.17)	(0.17)	(0.18)	(0.18)	(0.16)	(0.16)	(0.17)	(0.16)	(0.20)	(0.19)
adj. R^2	0.089	0.058	0.074	0.073	0.055	0.065	0.063	0.074	0.064	0.043	0.064
N	1332	1285	1329	1320	1280	1330	1323	1332	1332	1109	1332

Note: Unstandardized OLS regression estimates. Robust standard errors clustered on community in parentheses.
* $p < 0.05$, ** $p < 0.01$, *** $p < 0.001$ (two-sided tests).
Baseline categories: age (female), education (illeterate), clan (Hawiay).

Table A9: Table for Fig. 2 (S2) [With additional covariates]

	Acceptance	Acc.	Acc.	Acc.	Acc.	Acc.	Acc.	Acc.	Acc.	Acc.
Victim (violence)	0.20**									
	(0.07)									
Victim of GBV		0.33***								
		(0.08)								
Sickness (family)			0.18***							
			(0.04)							
Deaths (community)				0.10***						
				(0.03)						
Economic disruption					0.09***					
					(0.02)					
Trust (UN)						0.18***				
						(0.05)				
Community aid							0.12***			
							(0.02)			
Distrust West								-0.29		
								(0.14)		
West-facing Occ.									-0.10	
									(0.09)	
GBV (Community)										0.13*
										(0.05)
Male	-0.16**	-0.24***	-0.16**	-0.16**	-0.17**	-0.16**	-0.16**	-0.17**	-0.16**	-0.15**
	(0.06)	(0.06)	(0.05)	(0.05)	(0.05)	(0.05)	(0.05)	(0.05)	(0.05)	(0.05)
Primary	0.06	0.04	0.04	0.07	0.08	0.06	0.06	0.07	0.08	0.08
	(0.09)	(0.10)	(0.09)	(0.09)	(0.09)	(0.09)	(0.09)	(0.09)	(0.09)	(0.09)
Secondary	-0.18*	-0.12	-0.14	-0.13	-0.07	-0.12	-0.08	-0.12	-0.12	-0.14
	(0.09)	(0.09)	(0.08)	(0.08)	(0.08)	(0.08)	(0.08)	(0.08)	(0.08)	(0.08)
High school	-0.01	0.04	0.02	0.06	0.07	0.05	0.06	0.04	0.05	0.04
	(0.09)	(0.10)	(0.09)	(0.09)	(0.09)	(0.09)	(0.09)	(0.09)	(0.09)	(0.09)
University	0.08	0.19	0.08	0.09	0.14	0.11	0.12	0.12	0.12	0.09
	(0.16)	(0.15)	(0.14)	(0.15)	(0.15)	(0.15)	(0.14)	(0.15)	(0.14)	(0.15)
Only Madrassa	-0.23**	-0.26**	-0.15	-0.17*	-0.14	-0.13	-0.18*	-0.14	-0.18*	-0.16
	(0.08)	(0.08)	(0.08)	(0.08)	(0.08)	(0.08)	(0.07)	(0.08)	(0.08)	(0.08)
Darood	0.09	0.02	0.11	0.12	0.18	0.14	0.20*	0.10	0.13	0.12
	(0.11)	(0.12)	(0.10)	(0.10)	(0.09)	(0.11)	(0.09)	(0.10)	(0.11)	(0.10)
Ranweyn or Digil iyo mirifle	-0.07	-0.01	-0.06	-0.02	0.03	-0.05	0.06	-0.08	-0.05	-0.07
	(0.10)	(0.10)	(0.09)	(0.10)	(0.09)	(0.09)	(0.09)	(0.09)	(0.10)	(0.09)
Dir	-0.12	-0.13	-0.12	-0.10	0.00	-0.08	0.00	-0.09	-0.14	-0.14
	(0.17)	(0.19)	(0.17)	(0.17)	(0.15)	(0.18)	(0.16)	(0.17)	(0.17)	(0.17)
Other	-0.19	-0.19	-0.16	-0.17	-0.10	-0.12	-0.08	-0.16	-0.15	-0.18
	(0.17)	(0.18)	(0.15)	(0.17)	(0.16)	(0.16)	(0.15)	(0.16)	(0.17)	(0.16)
No. Children	-0.01	-0.02	-0.01	-0.01	-0.01	-0.01	-0.01	-0.01	-0.01	-0.01
	(0.01)	(0.01)	(0.01)	(0.01)	(0.01)	(0.01)	(0.01)	(0.01)	(0.01)	(0.01)
Age	0.01**	0.01**	0.01***	0.01***	0.01***	0.01***	0.01***	0.01***	0.01***	0.01**
	(0.00)	(0.00)	(0.00)	(0.00)	(0.00)	(0.00)	(0.00)	(0.00)	(0.00)	(0.00)
Constant	2.59***	2.74***	2.24***	2.31***	2.27***	2.02***	2.20***	2.67***	2.65***	2.35***
	(0.18)	(0.17)	(0.17)	(0.19)	(0.19)	(0.26)	(0.17)	(0.17)	(0.17)	(0.23)
adj. R^2	0.145	0.195	0.168	0.137	0.148	0.154	0.175	0.133	0.120	0.126
N	608	463	632	618	632	631	633	633	633	627

Note: Unstandardized OLS regression estimates. Robust standard errors clustered on community in parentheses.
* $p < 0.05$, ** $p < 0.01$, *** $p < 0.001$ (two-sided tests).
Baseline categories: age (female), education (illeterate), clan (Hawiay).

G Conditional Effects of Al-Shabaab Endorsement

This section explores the heterogeneous effects of the Al-Shabaab endorsement treatment by conditioning the treatment effects on exposure to violence (0 = no, 1 = yes, M = .52), exposure to GBV (0 = no, 1 = yes; M = .37), a measure of exposure to Al-Shabaab violence (having children abducted by Al-Shabaab; 1 = yes, 2 = no, M = 1.71), and on agreement with the statement that violence is necessary for Al-Shabaab to achieve its goals (0 = disagree, 1 = agree, M = .62).[1]

Table A10: Conditional Treatment Effects of Al-Shabaab Endorsement (R2)

	Vaccine Accept	Vaccine Accept	Vaccine Accept	Vaccine Accept
Al-Shabaab Endorsement	-0.55***	-0.54***	-0.47**	-0.56**
	(0.12)	(0.12)	(0.11)	(0.15)
Victim (violence)	0.14			
	(0.12)			
AS Endorsement × Victim (violence)	0.39*			
	(0.15)			
Victim of GBV		0.19		
		(0.16)		
AS Endorsement × Victim (GBV)		0.51*		
		(0.17)		
Children Abducted by AS (Yes)			-0.22	
			(0.13)	
AS Endorsement × Abduction			0.44*	
			(0.20)	
AS Violence Necessary				-0.56***
				(0.07)
AS Endorsement × Violence Necessary				0.30
				(0.17)
Constant	2.79***	2.87***	2.95***	3.27***
	(0.16)	(0.16)	(0.15)	(0.15)
adj. R^2	0.117	0.142	0.058	0.102
N	253	201	265	265

Note: Unstandardized OLS regression estimates. Robust standard errors clustered on community in parentheses.
* $p < 0.05$, ** $p < 0.01$, *** $p < 0.001$ (two-sided tests).

To ease interpretation, fig. A5 below shows the estimated marginal effects of the AS endorsement, conditional on these measures. Panel A shows that the AS endorsement produced a substantial negative effect on vaccine receptivity among respondents who had not been exposed to violence, but not among respondents who had been exposed to violence. As shown in table A10, this difference in marginal effects is significant (95% CI[.06, .70]; p = .024). Similarly, panel B shows that the marginal effect of the AS endorsement is negative and significant among those who have not been exposed to GBV, while it is not distinguishable from zero among victims of GBV. The difference in marginal effects is .51 (95% CI [.14, .87]; p = .011). In a similar vein, panel C shows that respondents who have had their children abducted by Al-Shabaab did not react as negatively to the AS endorsement as did respondents who had not had children abducted (difference in marginal effects = .44; 95% CI [.005, .86]; p = .048). Panels A-C all concern the effects of Al-Shabaab

[1]Answers to the statement that violence is necessary to Al-Shabaab to achieve its goals are given on a four-point scale. Here, we collapse these into a dichotomous indicator of agreement. Note that the findings using this measure differs slightly from the ordinal measure. When using the latter, the marginal effect of the AS treatment for respondents who ”agree somewhat” with the statement is insignificant, whereas the three other conditional treatment effects are negative and significant.

endorsements on vaccine receptivity, conditional on exposure to violence. In all three cases, an endorsement by Al-Shabaab decreased vaccine acceptance among those who had not been exposed to such violence, but not among those who had. Finally, panel D shows that an AS endorsement reduced vaccine receptivity among those who disagreed with the statement that violence is necessary for Al-Shabaab to achieve its goals and those who agreed with the statement. Although the negative effect of an AS endorsement is greater among respondents who disagree with the statement, this difference is not significant at conventional levels (difference in marginal effects = .29; 95% CI [-.06, .66]; $p = .102$). Taken together, the findings in this section suggest that exposure to violence may attenuate the negative reaction to Al-Shabaab vaccine endorsements.

Figure A5: Marginal Effects of AS Endorsement, conditional on exposure to violence

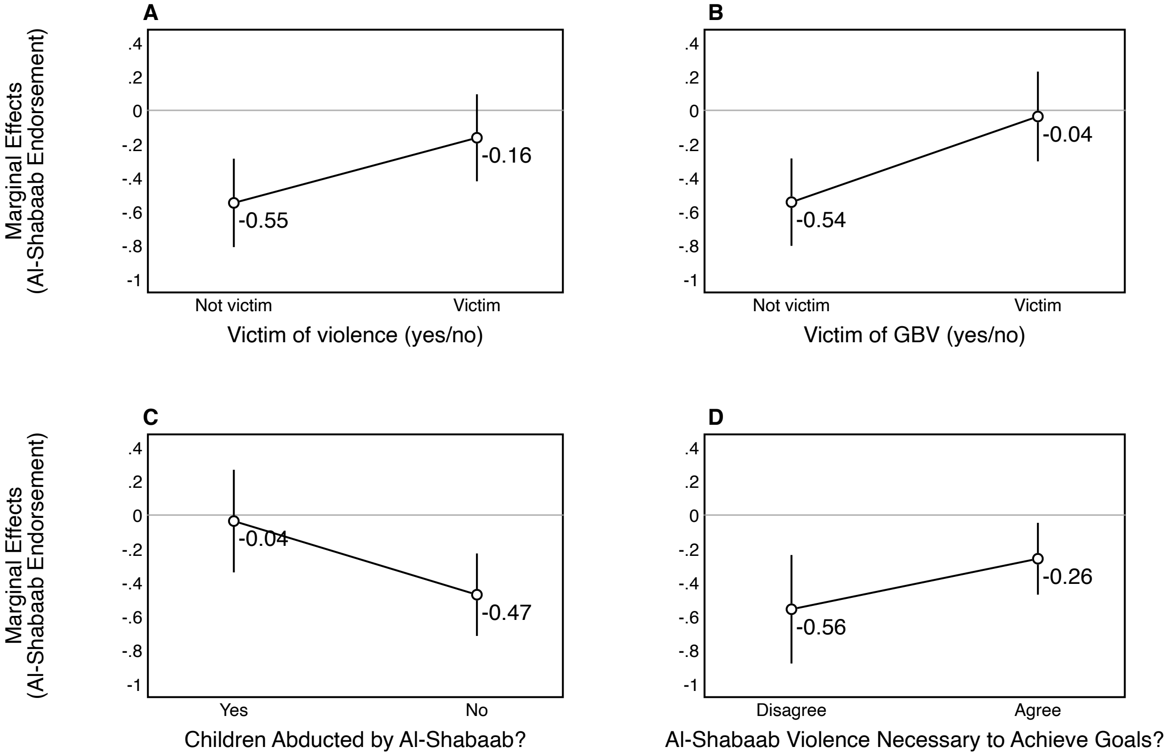

Note: Estimates based on unstandardized OLS regressions with city fixed effects. Whiskers are 95% confidence intervals based on robust standard errors clustered on communities. * $p < 0.05$, ** $p < 0.01$, *** $p < 0.001$ (two-sided tests).

H Conditional Effects of Traditional Authorities Endorsement

In this section, we explore the potential moderating effects of trust on the effects of vaccine endorsements by customary authorities (religious leaders and clan leaders). We rely on two pre-treatment measures on trust from survey round 2; generalized trust (0 = low trust, 1 = high trust, M = , SD =) and trust in local government authorities (0 = low trust, 1 = high trust, M = , SD =). The findings are shown in table A11 below, and fig. A6 displays the marginal effects of customary authority endorsements among high and low-trusting individuals.

Table A11: Conditional Treatment Effects of Traditional Authority Endorsement (R2)

	Vaccine Acceptance	Vaccine Acceptance
Traditional Authority Endorsement	-0.09	0.23
	(0.10)	(0.36)
Generalized trust	-0.25*	
	(0.08)	
TA Endorsement × Generalized trust	0.16	
	(0.11)	
Trust in local government		0.49
		(0.28)
TA Endorsement × Trust in local government		-0.31
		(0.38)
Constant	3.02***	2.52***
	(0.14)	(0.31)
adj. R^2	0.017	0.015
N	264	267

Note: Unstandardized OLS regression estimates. Robust standard errors clustered on community in parentheses.
* $p < 0.05$, ** $p < 0.01$, *** $p < 0.001$ (two-sided tests).

Table A11 shows that respondents with higher generalized trust (column 1) were less receptive of Covid-19 vaccination in baseline compared to low-trusting individuals ($\hat{\beta}$ = -0.25; 95% CI [-.42, -.07]; $p = .01$). Among individuals with low generalized trust, the effect of a TA endorsement is not statistically distinguishable from zero. The table also shows that the interaction term (TA endorsement × generalized trust) is positive ($\hat{\beta}$ = 0.16; 95% CI [-.07, .39]) but insignificant (p = 0.16). In contrast, column 2 shows that individuals with low trust in local government less receptive to the vaccine. This difference, however, is insignificant. Also in contrast to generalized trust, the table shows that the TA endorsement failed to produce a non-zero effect among low-trusting individuals ($\hat{\beta} = .23$; 95% CI [-.55, 1.01]; $p = .53$). Similar to column 1, the table shows that the interaction term is insignificant.

Fig. A6 below displays the estimated marginal effects of an endorsement by customary authorities, conditional on the individuals' generalized trust (panel a) and trust in local government (panel b). As shown, none of the marginal effect sizes are substantial or reach statistical significance. These findings substantiate our interpretation that stated trust is not a necessarily a meaningful predictor of vaccine receptivity in the context of Somalia.

Figure A6: Marginal Effects of TA Endorsement, Conditional on Trust

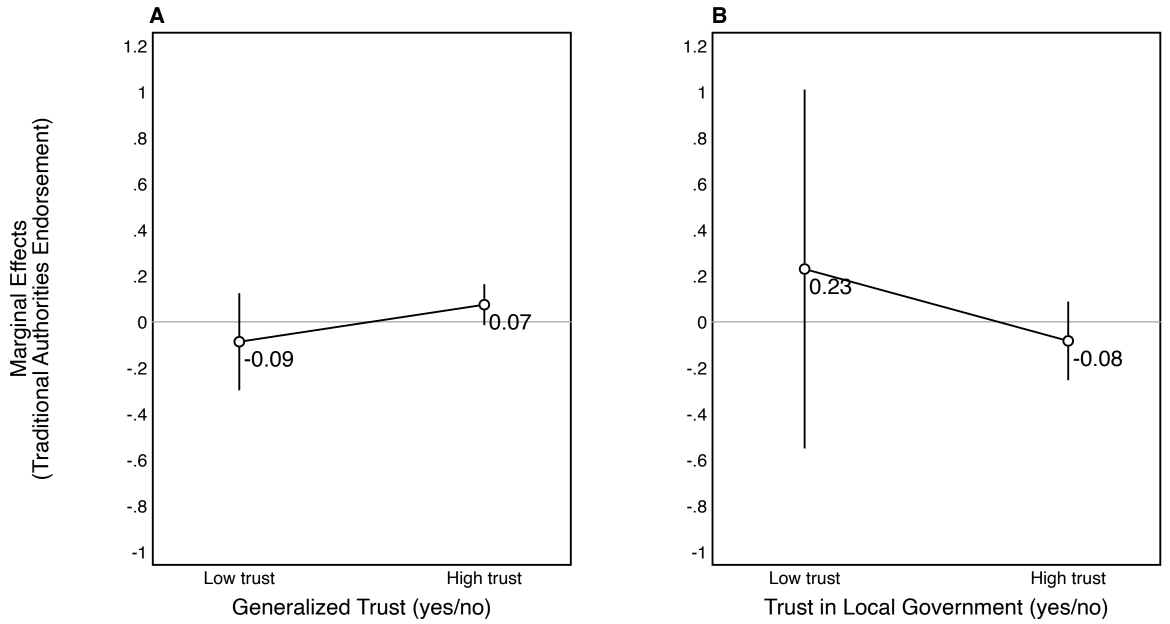

Note: Estimates based on unstandardized OLS regressions with city fixed effects. Whiskers are 95% confidence intervals based on robust standard errors clustered on communities. * $p < 0.05$, ** $p < 0.01$, *** $p < 0.001$ (two-sided tests).

I Estimating Within-Respondent Changes

Figure A7 displays the estimated correlations between within-respondent changes in predictors (wave 3 - wave 2) and vaccination status (dichotomous measure) in survey 3 using OLS regression functions with city fixed effects and standard errors clustered at the community level.

Figure A7: Within-Respondent Changes in Covariates Predict Vaccination Status

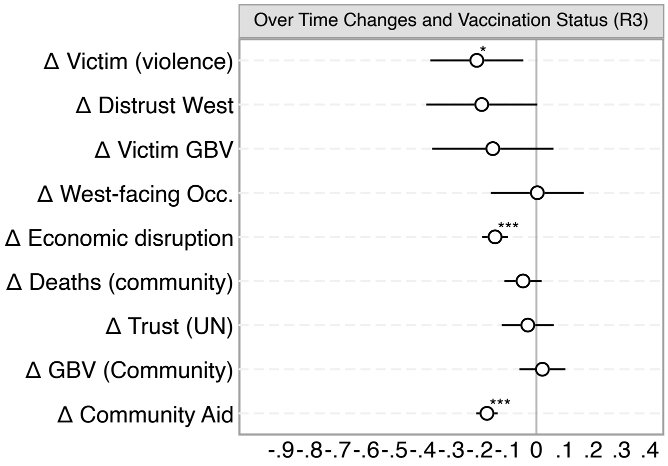

Note: Estimates based on unstandardized OLS regressions with city fixed effects. Whiskers are 95% confidence intervals based on robust standard errors clustered on communities. * $p < 0.05$, ** $p < 0.01$, *** $p < 0.001$ (two-sided tests).

J The Effect of Endorsements on Vaccine Take-up

This section examines the effects of endorsements in survey round 2 on vaccination status in survey round 3, using both the dichotomous measure (0 = not vaccinated, 1 = at least one dose) and the continuous measure (0 = no vaccinated, 1 = one dose, 2 = two doses, 3 = two doses + booster dose). Table A12 shows the estimated average treatment effects using unstandardized OLS regressions with city fixed effects and robust standard errors clustered on communities. As the findings show, our endorsement experiment in survey round 2 did not cause any increases or reduction in vaccine take-up in survey round 3.

Table A12: Effect of Endorsements (R2) on Vaccine Status (R3)

	Vaccine Status (dichotomous)	Vaccine Status (continuous)
Government Endorsement	-0.06	-0.11
	(0.06)	(0.10)
Trad. Authorities Endorsement	-0.04	0.00
	(0.05)	(0.09)
Int. Organizations Endorsement	-0.02	-0.02
	(0.05)	(0.09)
Al-Shabaab Endorsement	-0.02	0.00
	(0.08)	(0.14)
Constant	0.41*	0.43*
	(0.14)	(0.15)
City Fixed Effects	✓	✓
adj. R^2	0.022	0.023
N	491	491

Note: Unstandardized OLS regression estimates. Robust standard errors clustered on community in parentheses.
* $p < 0.05$, ** $p < 0.01$, *** $p < 0.001$ (two-sided tests).

K Additional Sampling Information

K.1 Script for Enumerators

Good morning / Good Afternoon / Good Evening. As you know we have been coming to your community to carry out sessions related to peace, security and health. Recently we have also been in your community providing awareness against the current COVID-19 Virus health pandemic. However, today we are here to collect your phone number for a survey that will be carried out by UNDP ROL- JPP project in the next 2-4 weeks. The phone number that will call you is: [Redacted]

- We would like to take this opportunity to kindly request if you will be willing to provide us with your phone number for the survey we will be doing in the next few weeks, which is voluntary. Even if you agree to provide us with your phone number for the survey, you have the right to withdraw at any point during the survey, for any reason.
- This is a no deception study and has been approved as such by the Ministry of Justice Somalia; everything you are told will be accurate to the best of our knowledge.
- If you would like to contact a Principal Investigator of the study to discuss this research, please e-mail Prabin Khadka at [Redacted] or message him on WhatsApp at [Redacted].
- Please indicate that you would like to provide your consent to give us your cell number to participate in the study by saying “yes”. If you would not like to participate in the study, please say “no”.

K.2 Generalizability- Comparing Our Study Sample with the World Bank 2021 Telephone Survey

The World Bank conducted a nationwide COVID-19 Somali High-Frequency Phone Survey (SHFPS) of households to track the socioeconomic effects of the pandemic. The aim of this survey, part of a global initiative, was to address various key areas such as COVID-19 knowledge, preventive actions taken, economic activities, income sources, access to essentials, dealing with shocks, and access to social assistance.

The survey was carried out in two rounds using a random digit dialing protocol. Round 1 of the COVID-19 SHFPS was implemented between June and July 2020 (2,811 households :1,735 urban households, 611 rural households, 435 nomadic households, and 30 IDP households in settlements). The sample of 2,811 households was contacted using a random digit dialing protocol. Round 2 of the COVID-19 SHFPS was implemented in January 2021. A total of 1,756 households were surveyed (738 urban households, 647 rural households, 309 nomadic households, and 62 IDP households in settlements). Of the 1,756 households, 91 percent were successfully re-contacted from Round 1, with the remainder reached via random digit dialing.

We only use Round 2 of the WB phone survey for our comparison tables below for three reasons: First, Round 2 of WB survey is a panel of Round 1 WB survey so demographics in Rnd 2 WB survey are also the same as Rnd 1 WB survey. Second, Round 2 WB survey coincides with our Round 1 phone survey as both surveys were carried out exactly in Jan 2021.Finally, Round 2 asks respondents “if vaccine was available, would they take it?”. Round 1 WB survey does not have any questions related to COVID-19 vaccination.

Documentation of the survey, including the questionnaire, data and reports, can be found here:link.

K.3 Data Comparison with World Bank Survey

In this Section, we compare our sample to the sample contained in the second round of the COVID-19 Somali High-Frequency Phone Survey (SHFPS) [6]. The SHFPS focused on evaluating the socio-economic impact of COVID-19 on communities in Somalia. Data for the second round of the SHFPS was collected in January 2021 and thus provides a particularly strong point of comparison for the first wave of our survey (also conducted in January of 2021), as well as our second wave (conducted in August of 2021).[2] The SHFPS was designed with the aim of obtaining a representative national sample of Somali respondents and included individuals from across different regions (Jubaland, South West, HirShabelle, Galmudug, Puntland, Somaliland, and Banadir Regional Administration) and types of populations (urban, rural, nomads, and internally displaced populations). Round 2 of the survey was conducted with 1,756 households and was done using Computer Assisted Telephone Interview (CATI) methods.

In Tables A13 and A14, we compare our sample to, respectively, the full SHFPS sample and the SHFPS sample restricted to the same seven urban areas in our study. A few observations are worth note. First, we observe no statistically distinguishable difference between our sample and the overall or restricted SHFPS samples as regards our central outcome of interest: vaccine receptivity. Second, a comparison of the overall and restricted SHFPS samples suggests that on key demographics and outcomes, the seven cities we sample are not substantially different from the rest of Somalia. These findings are encouraging, insofar as they provide support for the notion that our findings regarding vaccine receptivity would generalize not only to the specific cities we sample, but potentially to all of Somalia (or at least that portion represented in the SHFPS sample).[3]

Third, we find no differences between our sample and the overall or restricted samples as regards respondent age, and only differences for gender for wave 1 of our survey – after which stage we adjusted to ensure more male respondents, as discussed in the Methods Section of the main text. Fourth, we observe that our sample reports higher levels of literacy than both the overall and restricted SHFPS samples.[4] Importantly, however, as we discuss and demonstrate in the paper and SI, these differences are unlikely to bias our findings or undercut their generalizability: to this end, we show that results are robust to within-respondent analyses and controlling for respondent demographics (including gender and education); further, as discussed above, we find that our central outcome of interest (vaccine receptivity) is similar across samples.

In sum, then, our analysis indicates that our findings are likely to generalize both to the seven cities sampled and to larger portions of Somalia.

[2]We only include the first two waves of our survey as a comparison firstly because they are more proximate in terms of survey timing and secondly, because we can use our measure of vaccine receptivity in these waves as a comparison point (in contrast, we only measured vaccine take-up in round 3).

[3]As we note in the main text, due to both safety and logistical concerns, rural and Al-Shabaab controlled areas are undersampled both in our study and likely the SHFPS.

[4]We can only speculate as to why this might be the case; it is possible, for instance, that more educated individuals were more comfortable speaking with and providing their information to our enumerators during our phone listing exercise.

Table A13: Comparing Our Study Sample (Round 1 and Round 2) with the World Bank Phone Survey[6]: **Entire Sample**

**Variable by Study Sample**	Mean	S.D.	Min	Max	*N*	Mean Difference
Male						
World Bank Round 2 (January 2021)	0.50	0.50	0	1	1,756	
Our Study Sample Round 1 (January 2021)	0.39	0.48	0	1	1,663	
Our Study Sample Round 2 (August 2021)	0.50	0.50	0	1	880	
Two-sample t test: WB and Our Sample Round 1						0.11***
						(0.01)
Two-sample t test: WB and Our Sample Round 2						0.00
						(0.02)
Age						
World Bank Round 2 (January 2021)	38.09	13.67	18	97	1,754	
Our Study Sample Round 1 (January 2021)	38.10	13.61	18	90	1,663	
Our Study Sample Round 2 (August 2021)	37.87	12.85	18	90	880	
Two-sided t test: WB and Our Sample Round 1						-0.01
						(0.46)
Two-sided t test: WB and Our Sample Round 2						0.21
						(0.55)
Education: Literate						
World Bank Round 2 (January 2021)	0.73	0.43	0	1	1,665	
Our Study Sample Round 1 (January 2021)	0.87	0.32	0	1	1,663	
Our Study Sample Round 2 (August 2021)	0.84	0.36	0	1	880	
Two-sided t test: WB and Our Sample Round 1						-0.13***
						(0.01)
Two-sided t test: WB and Our Sample Round 2						-0.10***
						(0.01)
Willingness to be Vaccinated (COVID-19)						
World Bank Round 2 (January 2021)	0.90	0.29	0	1	1,747	
Our Study Sample Round 1 (January 2021)	0.90	0.30	0	1	334	
Our Study Sample Round 2 (August 2021)	0.93	0.24	0	1	176	
Two-sided t test: WB and Our Sample Round 1						0.00
						(0.01)
Two-sided t test: WB and Our Sample Round 2						-0.03
						(0.02)

Note: Table shows comparison between the World Bank telephone COVID-19 survey entire Round 2 sample and our study sample (Rounds 1 and 2). Two-sided t-tests between WB and Round 1 and between WB and Round 2 also presented.

Table A14: Comparing Our Study Sample (Round 1 and Round 2) with the World Bank Phone Survey[6]: **Only Overlapping Urban Seven Cities Sample**

**Variable by Study Sample**	Mean	S.D.	Min	Max	*N*	Mean Difference
Male						
World Bank Round 2 (January 2021)	0.47	0.50	0	1	304	
Our Study Sample Round 1 (January 2021)	0.38	0.48	0	1	1,663	
Our Study Sample Round 2 (August 2021)	0.50	0.50	0	1	880	
Two-sample t test: WB and Our Sample Round 1						0.08***
						(0.03)
Two-sample t test: WB and Our Sample Round 2						-0.02
						(0.03)
Age						
World Bank Round 2 (January 2021)	39.04	13.47	18	97	304	
Our Study Sample Round 1 (January 2021)	38.10	13.61	18	90	1,663	
Our Study Sample Round 2 (August 2021)	37.87	12.85	18	90	880	
Two-sided t test: WB and Our Sample Round 1						0.93
						(0.84)
Two-sided t test: WB and Our Sample Round 2						1.16
						(0.86)
Education: Literate						
World Bank Round 2 (January 2021)	0.78	0.42	0	1	293	
Our Study Sample Round 1 (January 2021)	0.87	0.32	0	1	1,663	
Our Study Sample Round 2 (August 2021)	0.84	0.36	0	1	880	
Two-sided t test: WB and Our Sample Round 1						-0.09***
						(0.02)
Two-sided t test: WB and Our Sample Round 2						-0.06***
						(0.02)
Willingness to be Vaccinated (COVID-19)						
World Bank Round 2 (January 2021)	0.93	0.25	0	1	303	
Our Study Sample Round 1 (January 2021)	0.90	0.30	0	1	334	
Our Study Sample Round 2 (August 2021)	0.93	0.24	0	1	176	
Two-sided t test: WB and Our Sample Round 1						0.03
						(0.02)
Two-sided t test: WB and Our Sample Round 2						-0.00
						(0.02)

Note: Table shows comparison between the World Bank telephone COVID-19 survey Round 2 limited to only urban residents in the seven cities in our sample, and our study samples (Rounds 1 and 2). Two-sided t-tests between WB and Round 1 and between WB and Round 2 also presented.

L Attrition across demographic groups and survey rounds

Table R1 displays the sample composition across a range of demographic characteristics across the three survey waves. Note that for the second round of data collection, we deliberately opted to obtain a more gender balanced sample. In the first survey round, we obtained a sample with more women than men due to their greater availability when contacted. To increase gender balance, we therefore sampled more men from survey round on in the two subsequent waves (rounds two and three). Aside from this change in sample composition, our sample is very balanced across survey rounds. The only noteworthy exceptions are on the share of respondents, who report being unemployed. Here, we observe a change from 46% in round one to about 38% in round two. Similarly, we see that the share of illiterate respondents is slightly larger in round 2. To account for such differences, we perform a series of additional analyses. First, we restrict the sample to only include respondents that are in both rounds one and two. Second, we perform *within-respondent* analyses, examining how changes within each respondent is associated with vaccine receptivity. Both sets of results are in the supplementary material. As these analyses yield similar findings, it effectively rules out the explanation that the observed differences in associations predictors and receptivity between rounds one and two are caused by differences in sample composition.

Table A15: Demographic Composition of Sample across Rounds

	**Round 1**		**Round 2**		**Round 3**	
	**M**	**SD**	**M**	**SD**	**M**	**SD**
Male	.38.7	.48	.5	.5	.49	.5
Age	38.1	13.6	37.8	12.8	37.2	12.6
Kismayo	.14	.35	.14	.34	.11	.32
Hudur	.16	.36	.16	.36	.16	.37
Baidoa	.14	.35	.15	.35	.17	.38
Galkayo	.14	.34	.13	.33	.11	.32
Beledwyne	.15	.35	.15	.35	.16	.38
Dhusanareb	.14	.35	.15	.35	.12	.32
Jowhaar	.13	.33	.14	.34	.13	.33
No. Children	6.06	3.13	6.15	3.49	N/A	N/A
Married	.805	.39	.813	.39	N/A	N/A

M Anonymized Photographs: Phone Number Collection

To see anonymized photographs of the data collection process, please follow this link.

References

[1] GPI, T. Global peace index. *Institute for Economics & Peace* (2019).

[2] Sundberg, R. & Melander, E. Introducing the ucdp georeferenced event dataset. *Journal of Peace Research* **50**, 523–532 (2013).

[3] Raleigh, C., Linke, r., Hegre, H. & Karlsen, J. Introducing acled: An armed conflict location and event dataset. *Journal of peace research* **47**, 651–660 (2010).

[4] Lazarus, J. V. *et al.* A global survey of potential acceptance of a covid-19 vaccine. *Nature medicine* **27**, 225–228 (2021).

[5] Solís Arce, J. S. *et al.* Covid-19 vaccine acceptance and hesitancy in low-and middle-income countries. *Nature medicine* **27**, 1385–1394 (2021).

[6] Karamba, W. Covid-19 somali high-frequency phone survey (shfps). *World Bank* (2021). URL `https://doi.org/10.48529/1mdv-qg24`.
